# PAX3::FOXO1-Targeting PROTAC Induces Myogenic Differentiation of Fusion-Positive Rhabdomyosarcoma Cells

**DOI:** 10.3390/cancers18152375

**Published:** 2026-07-23

**Authors:** Nikola Knoll, Kayra Somay, Purushottam B. Tiwari, Emre Deniz, Jeffrey S. S. K. Formen, Isabel Frye, Eryn Nelson, Christian Wolf, Jeffrey A. Toretsky, Aykut Üren

**Affiliations:** 1 Lombardi Comprehensive Cancer Center, Georgetown University Medical Center, Washington, DC 20057, USA; 2 Chemistry Department, Georgetown University, Washington, DC 20057, USA

**Keywords:** pediatric sarcoma, fusion-positive rhabdomyosarcoma, PAX3::FOXO1, oncogenic fusion protein, PROTAC

## Abstract

**Background/Objectives:** Fusion-positive rhabdomyosarcoma (FP-RMS) is characterized by the presence of tumor-specific chromosomal translocation products, most commonly PAX3::FOXO1, and typically results in lower survival rates compared to fusion-negative RMS cases. PAX3::FOXO1 plays a critical role in FP-RMS oncogenesis in both tumor initiation and maintenance, making it an excellent target for therapeutic intervention in FP-RMS. **Methods:** We created Proteolysis Targeting Chimeras (PROTACs) by combining PAX3::FOXO1-binding small molecules with E3 ligase recruiters for cereblon (CRBN) or S-Phase Kinase Associated Protein 1 (SKP1). **Results:** The PROTACs achieved up to 70% degradation of the endogenous PAX3::FOXO1 protein in FP-RMS cell lines in a concentration-, time-, and proteasome-dependent manner. Moreover, the PROTAC-mediated targeted degradation of PAX3::FOXO1 in FP-RMS cells deregulated the endogenous PAX3::FOXO1 gene expression signature and induced myogenic differentiation. Importantly, treatment of FP-RMS cells with PAX3::FOXO1-PROTACs synergized with vincristine treatment and impaired >80% of anchorage-independent growth in soft agar. **Conclusions:** Taken together, we demonstrate the proof of principle of PROTACs targeting the oncogenic fusion protein PAX3::FOXO1 in FP-RMS cells. The PROTACs created in this study will not only be useful tools in studying PAX3::FOXO1 biology in laboratory models but could also serve as molecular scaffolds for designing clinical-grade molecules to assess the therapeutic potential of PAX3::FOXO1-targeting PROTACs in FP-RMS patients.

## Simple Summary

Rhabdomyosarcoma (RMS) is the most common soft tissue cancer in young children and young adults. A subset of RMS tumors, called fusion-positive (FP-) RMS, harbors a genetic alteration resulting in a fusion protein called PAX3::FOXO1 that is only present in tumor cells and drives the disease. FP-RMS patients have not seen new effective treatment strategies in over 40 years and have an unacceptably low 5-year survival rate of 30%. Thus, the aim of this study was to design a new drug that would effectively target the tumor-specific PAX3::FOXO1 protein in FP-RMS. Therefore, we designed Proteolysis Targeting Chimeras (PROTACs) to degrade and eliminate the PAX3::FOXO1 protein in FP-RMS cells, which demonstrated effective disruption of the PAX3::FOXO1 protein and its function. The compounds that we describe and characterize here can serve as tools to study FP-RMS biology and be used to design clinical-grade drugs for the treatment of FP-RMS in the future.

## 1. Introduction

Rhabdomyosarcoma (RMS) is the most common soft tissue tumor, which primarily occurs in young children [1,2,3]. The five-year survival rate of patients with localized disease and a favorable histological subtype is approximately 70% [4]. However, patients with metastatic disease and tumors of unfavorable histological subtypes have a dismal five-year survival rate of 30% [5,6]. Unfavorable histological subtypes of RMS are characterized by the presence of tumor-specific chromosomal translocations giving rise to fusion proteins. The most common fusion protein, PAX3::FOXO1, arises from a t(2;13) chromosomal translocation and harbors the two N-terminal DNA-binding domains of PAX3 and the C-terminal transactivation domain of FOXO1 [7,8,9,10]. The transcriptional activity of PAX3::FOXO1 is 10–100-fold higher than that of the wild type (WT) PAX3 [11,12,13]. Even though there are other RMS-associated fusion proteins, PAX3::FOXO1 is the defining feature of fusion-positive (FP-) RMS and is critical in both tumor development and chemotherapy response [14,15,16,17,18,19,20].

PAX3::FOXO1 is only expressed in tumor cells, and its oncogenic function drives the disease [14,15,16,17,18]; thus, it represents an attractive therapeutic target. Despite decades of research, no new effective treatments have reached FP-RMS patients in over 40 years, which is reflected in the persistently poor survival rates that have remained largely unchanged. Temsirolimus is the only drug that has been introduced in the last four decades but provided a limited overall survival benefit [21]. Several research groups have aimed to phenocopy PAX3::FOXO1 loss by pharmaceutical approaches or indirectly targeted the PAX3::FOXO1 function [20,22,23,24,25,26,27,28,29,30], which highlighted the therapeutic potential of impairing PAX3::FOXO1 activity in FP-RMS. However, none of the existing approaches specifically and directly target the fusion protein. Currently, there is no drug in clinical use that directly targets the PAX3::FOXO1 protein. We discovered piperacetazine as the first-in-class small molecule that can specifically bind to PAX3::FOXO1 and inhibit its transcriptional activity; however, it was not potent enough to be developed as a therapeutic [31].

It has been historically difficult to target transcription factors with conventional small molecule-based approaches. The recent advances in targeted protein degradation (TPD) provide unique opportunities to address some of the limitations of small molecule inhibitors. TPD modalities such as proteolysis-targeting chimeras (PROTACs), first described in 2001 [32], represent a promising new approach that expands the druggable proteome via its unique mechanism of action. PROTACs are heterobifunctional molecules that hijack the ubiquitin-proteasome system (UPS) by bringing the protein of interest (POI) into close proximity of an E3 ligase, which leads to ubiquitination and subsequent proteasomal degradation of the POI [33,34]. PROTACs follow event-driven pharmacology that does not require strong binding to target proteins. Therefore, PROTACs enable potent target degradation even with weak affinity between the PROTAC and target protein [35,36,37,38].

In this study, we designed PAX3::FOXO1-targeting PROTACs using PAX3::FOXO1 small molecule binders identified from our previously reported surface plasmon resonance (SPR) screen [31]. We further optimized the PROTAC design by recruiting the E3 ligase S-Phase Kinase Associated Protein 1 (SKP1) instead of cereblon (CRBN) due to its higher expression and dependency in FP-RMS cells. We characterized the SKP1-based PAX3::FOXO1 PROTAC for effective concentration-, time-, and proteasome-dependent degradation of the endogenous PAX3::FOXO1 protein in FP-RMS cells. Bulk RNA-sequencing and gene set enrichment analysis (GSEA) of PROTAC-treated FP-RMS cells demonstrated impairment of PAX3::FOXO1 target gene expression accompanied by myogenic differentiation. Importantly, the PRTOAC treatment synergized with vincristine treatment and impaired anchorage-independent growth in FP-RMS cells.

## 2. Materials and Methods

### 2.1. Chemicals

Unless otherwise stated, all chemicals were purchased from Sigma-Aldrich (St. Louis, MO, USA). **PFB-a** to **PFB-f** were prepared in-house; PROTACs were synthesized by WuXi AppTec (Tianjin) Co., Ltd. (Tianjin, China) and dissolved in DMSO at 10 mM. MG132 (MedChemExpress (Junction, NJ, USA), catalog no. HY-13259), MLN4924 (MedChemExpress catalog no. HY-70062), dTAG-47 (MedChemExpress, catalog no. HY-147098), and vincristine (Selleck Chemicals, Houston, TX, USA, catalog no. S9555) were purchased and dissolved in DMSO to 10 mM working stocks.

### 2.2. Synthesis and Characterization of the PFB Compounds


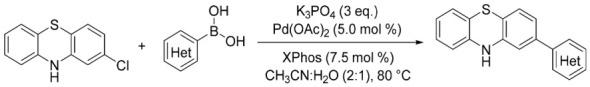


Step 1: 2-Chlorophenothiazine (1.0 mmol), K_3_PO_4_ (3.0 mmol), and boronic acid (1.5 mmol) were dissolved in 1 mL CH_3_CN/H_2_O (2:1) under a nitrogen atmosphere. The solution was heated to 40 °C, followed by the addition of Pd(OAc)_2_ (5.0 mol%) and XPhos (7.5 mol%). The reaction was stirred at 40 °C for 30 min, then heated to 80 °C and monitored by TLC. Upon completion, the reaction was cooled to room temperature and the CH_3_CN was removed under vacuum. The remaining crude mixture was extracted with CH_2_Cl_2_ (15 mL), dried over MgSO_4_, and concentrated under vacuum. The residue was purified by flash column chromatography using EtOAc:hexanes as the eluent.


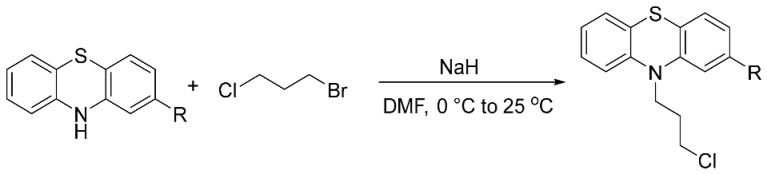


Step 2: Sodium hydride (9.0 mmol) was added to 3.0 mL anhydrous DMF under a nitrogen atmosphere, and the suspension was cooled to 0 °C. The phenothiazine (3.0 mmol) was added and stirred for 5 min, followed by the addition of 1-bromo-3-chloropropane (9.0 mmol). The solution was warmed to room temperature and monitored by TLC. Upon completion, the reaction was cooled to 0 °C, quenched with brine, extracted with EtOAc (30 mL), dried over Na_2_SO_4_, and concentrated under vacuum. The residue was purified by flash chromatography on silica gel using hexanes as the eluent.


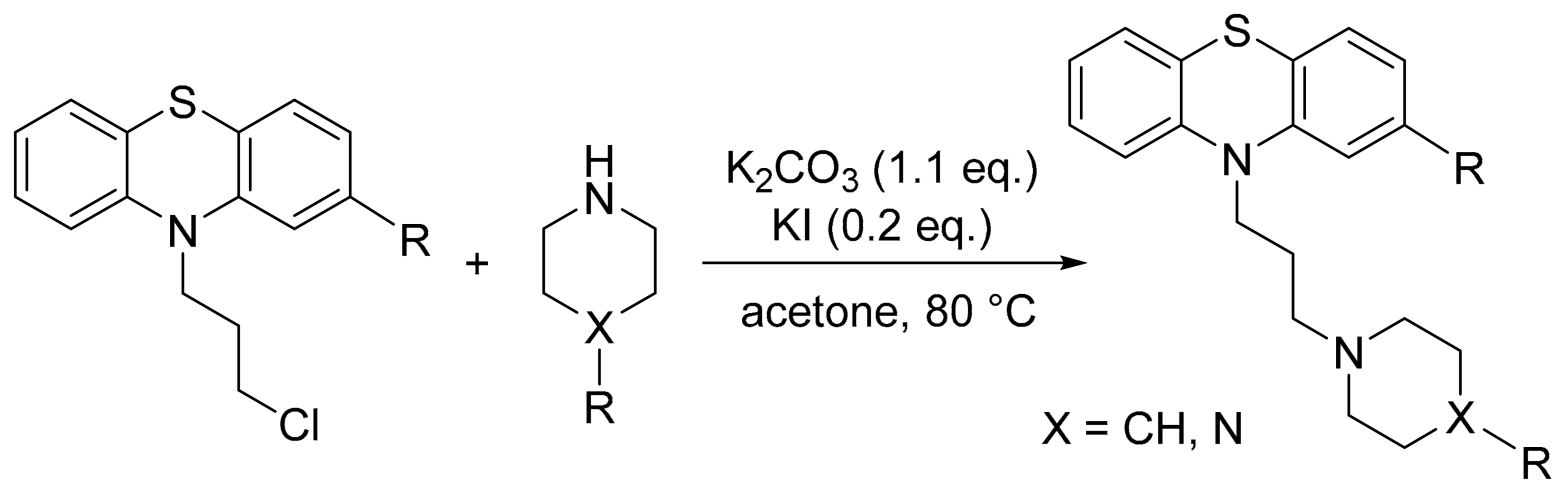


Step 3: The phenothiazine derivative (0.3 mmol), an amine (0.3 mmol), 1.1 equivalents of potassium carbonate, and potassium iodide (20 mol%) were combined in a pressure vessel with 1.0 mL acetone, heated to 80 °C, and the reaction was monitored by TLC. Upon completion, the crude reaction mixture was directly loaded on silica gel and purified by flash chromatography using MeOH/CH_2_Cl_2_ as the eluent. The resulting product was dissolved in CH_2_Cl_2_ followed by the addition of 1 M HCl (1 mL in diethyl ether). The supernatant was removed, and the precipitate was washed with ether and centrifuged (3 times). The solid was dissolved in MeOH, then concentrated under vacuum at 50 °C to yield the corresponding HCl salt.


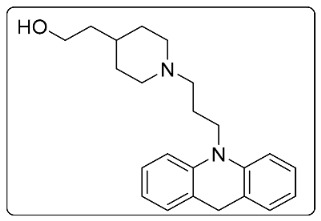


**2-(1-(3-(Acridin-10(9*H*)-yl)propyl)piperidin-4-yl)ethan-1-ol (PFB-a).**

Compound **PFB-a** was obtained from column purification using CH_2_Cl_2_/MeOH as mobile phase to give a white solid in 13% overall yield (45.5 mg, 0.13 mmol) following steps 2–3 of the general procedure described above using dihydroacridine instead of phenothiazine. ^1^H NMR (400 MHz, CD_3_OD) δ 7.16–7.06 (m, 4H), 6.95 (dd, *J* = 8.2, 1.1 Hz, 2H), 6.84 (ddd, *J* = 7.3, 7.3, 1.1 Hz, 2H), 3.93 (t, *J* = 7.2 Hz, 2H), 3.83 (s, 2H), 3.56 (t, *J* = 6.5 Hz, 2H), 3.10 (dt, *J* = 12.6, 3.4 Hz, 2H), 2.76–2.67 (m, 2H), 2.38–2.26 (m, 2H), 2.10–1.96 (m, 2H), 1.81–1.71 (m, 2H), 1.53 (m, 1H), 1.48–1.40 (m, 2H), 1.35–1.20 (m, 2H). ^13^C NMR (100 MHz, CD_3_OD) δ 142.0, 127.6, 126.6, 124.0, 120.3, 112.2, 58.8, 55.0, 53.2, 42.3, 38.3, 32.3, 31.2, 30.5, 22.1. HRMS (ESI-TOF) *m*/*z*: [M + H]^+^ calcd for C_23_H_30_N_2_O 351.2431, found 351.2430.


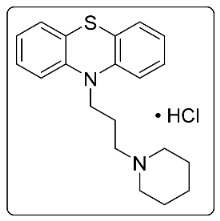


**10-(3-(Piperidin-1-yl)propyl)-10*H*-phenothiazine hydrochloride (PFB-b).**

Compound **PFB-b** was obtained from column purification using CH_2_Cl_2_/MeOH as mobile phase followed by precipitation with 1 M HCl in ether to give a yellow solid in 47% overall yield (17.0 mg, 0.05 mmol) following steps 2 and 3 of the general procedure described above. ^1^H NMR (400 MHz, CD_3_OD) δ 7.21 (ddd, *J* = 7.7, 7.7, 1.5 Hz, 2H), 7.15 (dd, *J* = 7.7, 1.5 Hz, 2H), 7.03 (dd, *J* = 8.2, 1.2 Hz, 2H), 6.95 (ddd, *J* = 7.5, 7.5, 1.2 Hz, 2H), 4.07 (t, *J* = 6.2 Hz, 2H), 3.50–3.31 (m, 2H), 3.21–3.12 (m, 2H), 3.09–2.67 (m, 2H), 2.24–2.12 (m, 2H), 1.91–1.37 (m, 6H). ^13^C NMR (100 MHz, CD_3_OD) δ 144.9, 127.4, 127.1, 125.9, 122.8, 115.8, 54.6, 53.0, 43.7, 22.7, 21.4, 21.1. HRMS (ESI-TOF) *m*/*z*: [M + H]^+^ calcd for C_20_H_24_N_2_S 325.1733, found 325.1733.


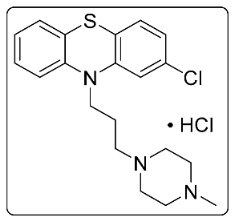


**2-Chloro-10-(3-(4-methylpiperazin-1-yl)propyl)-10*H*-phenothiazine hydrochloride (PFB-d).**

Compound **PFB-d** was obtained from column purification using CH_2_Cl_2_/MeOH as a mobile phase followed by precipitation with 1 M HCl in ether to give a yellow solid, 37% overall yield (134.3 mg, 0.36 mmol), following steps 2 and 3 of the general procedure described above. ^1^H NMR (400 MHz, CD_3_OD) δ 7.23 (ddd, *J* = 7.8, 7.8, 1.5 Hz, 1H), 7.15 (dd, *J* = 7.7, 1.5 Hz, 1H), 7.11–7.03 (m, 3H), 7.02–6.91 (m, 2H), 4.07 (t, *J* = 6.5 Hz, 2H), 3.98–3.39 (m, 8H), 3.39–3.30 (m, 2H), 2.96 (s, 3H), 2.31–2.19 (m, 2H). ^13^C NMR (100 MHz, cd_3_od) δ 146.44, 144.06, 133.30, 127.87, 127.60, 127.26, 125.50, 124.52, 123.24, 122.54, 116.20, 115.99, 54.31, 49.88, 48.50, 43.70, 41.88, 21.44. HRMS (ESI-TOF) *m*/*z*: [M + H]^+^ calcd for C_20_H_24_ClN_3_S 374.1452, found 374.1454.


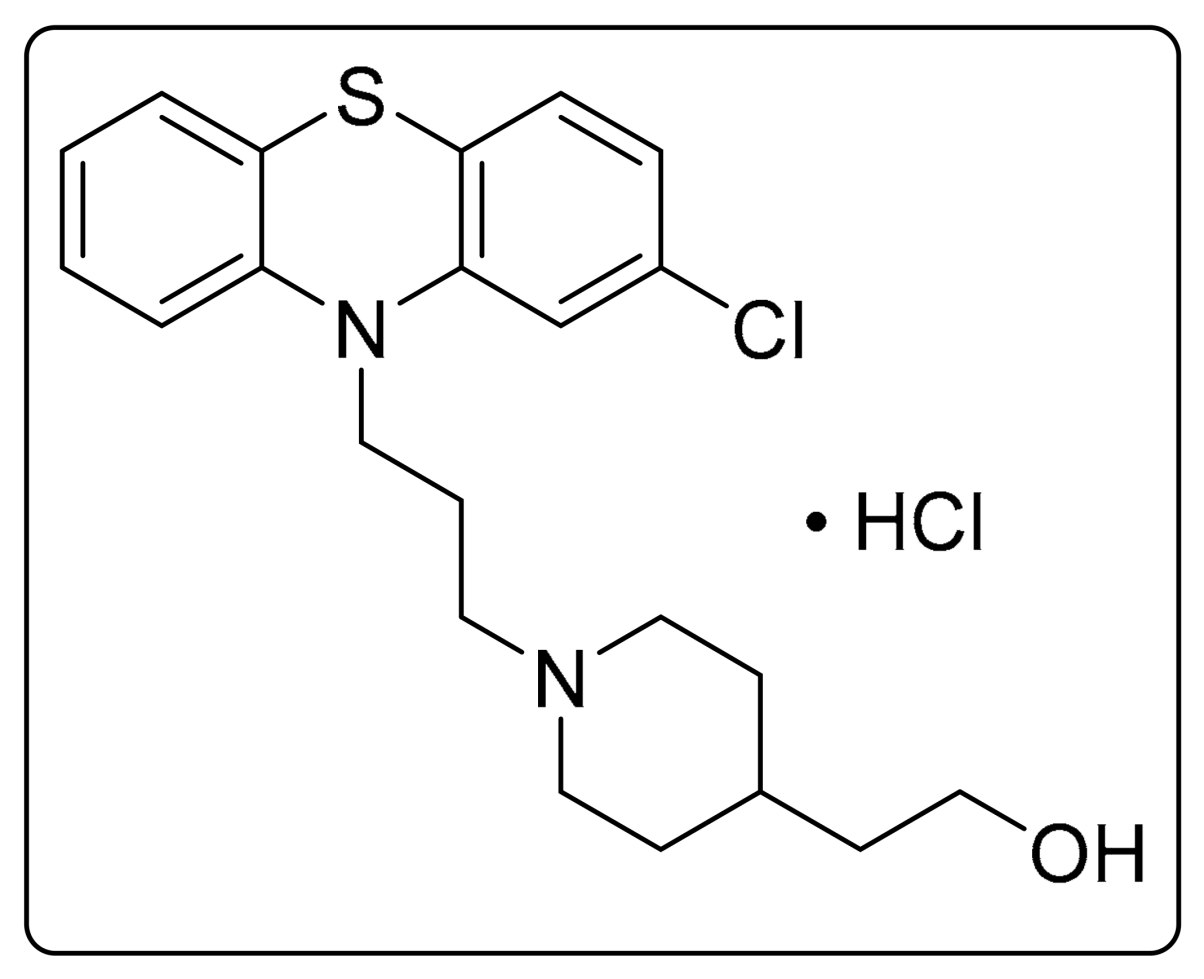


**2-(1-(3-(2-Chloro-10*H*-phenothiazin-10-yl)propyl)piperidin-4-yl)ethan-1-ol hydrochloride (PFB-e).**

Compound **PFB-e** was obtained from column purification using CH_2_Cl_2_/MeOH as mobile phase followed by precipitation with 1 M HCl in ether to give a yellow solid in 17% overall yield (149.8 mg, 0.37 mmol) following steps 2 and 3 of the general procedure described above. ^1^H NMR (400 MHz, CD_3_OD) δ 7.23 (ddd, *J* = 7.7, 7.7, 1.5 Hz, 1H), 7.15 (dd, *J* = 7.7, 1.5 Hz, 1H), 7.11–7.02 (m, 3H), 7.01–6.92 (m, 2H), 4.05 (t, *J* = 6.3 Hz, 2H), 3.57 (t, *J* = 6.4 Hz, 2H), 3.42–3.35 (m, 2H), 3.21–3.13 (m, 2H), 2.93–2.82 (m, 2H), 2.24–2.12 (m, 2H), 1.93–1.84 (m, 2H), 1.71 (m, 1H), 1.51–1.32 (m, 4H). ^13^C NMR (100 MHz, CD_3_OD) δ 146.5, 144.1, 133.3, 127.9, 127.6, 127.3, 125.6, 124.6, 123.3, 122.6, 116.3, 116.0, 58.5, 54.6, 52.9, 43.8, 37.8, 30.1, 29.3, 21.5. HRMS (ESI-TOF) *m*/*z*: [M + H]^+^ calcd for C_22_H_27_ClN_2_OS 403.1605, found 403.1605.


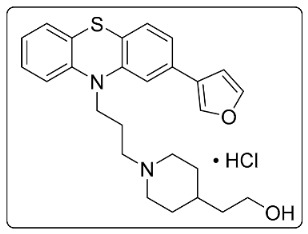


**2-(1-(3-(2-(Furan-3-yl)-10*H*-phenothiazin-10-yl)propyl)piperidin-4-yl)ethan-1-ol hydrochloride (PFB-f).**

Compound **PFB-f** was obtained from column purification using CH_2_Cl_2_/MeOH as mobile phase followed by precipitation with 1 M HCl in ether to give a yellow solid in 19% overall yield (79.2 mg, 0.18 mmol) following steps 1–3 of the general procedure described above. ^1^H NMR (400 MHz, CD_3_OD) δ 7.98 (s, 1H), 7.52 (d, *J* = 1.8 Hz, 1H), 7.24–7.07 (m, 5H), 7.03 (d, *J* = 8.1 Hz, 1H), 6.94 (dd, *J* = 7.5, 7.5 Hz, 1H), 6.82 (d, *J* = 1.9 Hz, 1H), 4.11 (t, *J* = 6.3 Hz, 2H), 3.54 (t, *J* = 6.4 Hz, 2H), 3.40–3.30 (m, 2H), 3.21–3.12 (m, 2H), 2.84–2.72 (m, 2H), 2.23–2.11 (m, 2H), 1.86–1.78 (m, 2H), 1.65 (m, 1H), 1.46–1.28 (m, 4H). ^13^C NMR (100 MHz, CD_3_OD) δ 145.5, 144.7, 143.8, 139.1, 132.3, 127.5, 127.5, 127.2, 125.9, 125.9, 124.2, 122.9, 120.3, 116.2, 113.2, 108.4, 58.6, 54.7, 52.9, 44.0, 37.8, 30.1, 29.2, 21.6. HRMS (ESI-TOF) *m*/*z*: [M + H]^+^ calcd for C_26_H_30_N_2_O_2_S 435.2101, found 435.2096.

### 2.3. Synthesis and Characterization of PFP-16

#### 2.3.1. Synthesis of the Phenoxazine Moiety and Final Coupling Step

Following the synthetic methodology described above, phenoxazine was derivatized with 1-bromo-3-chloropropane toward chloride **2,** which was directly used to prepare the primary alcohol **3**. Esterification with compound **4**, which was prepared separately, was achieved using EDCI (1-ethyl-3-(3-dimethylaminopropyl)carbodiimide). The structure of **PFP-16** was verified by ^1^H NMR and MS analysis.


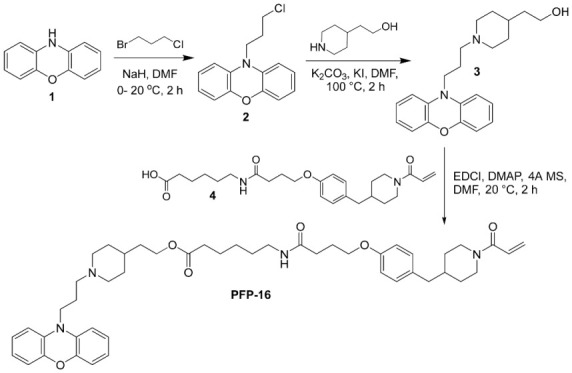


^1^H NMR spectrum of **PFP-16** in d_6_-DMSO:


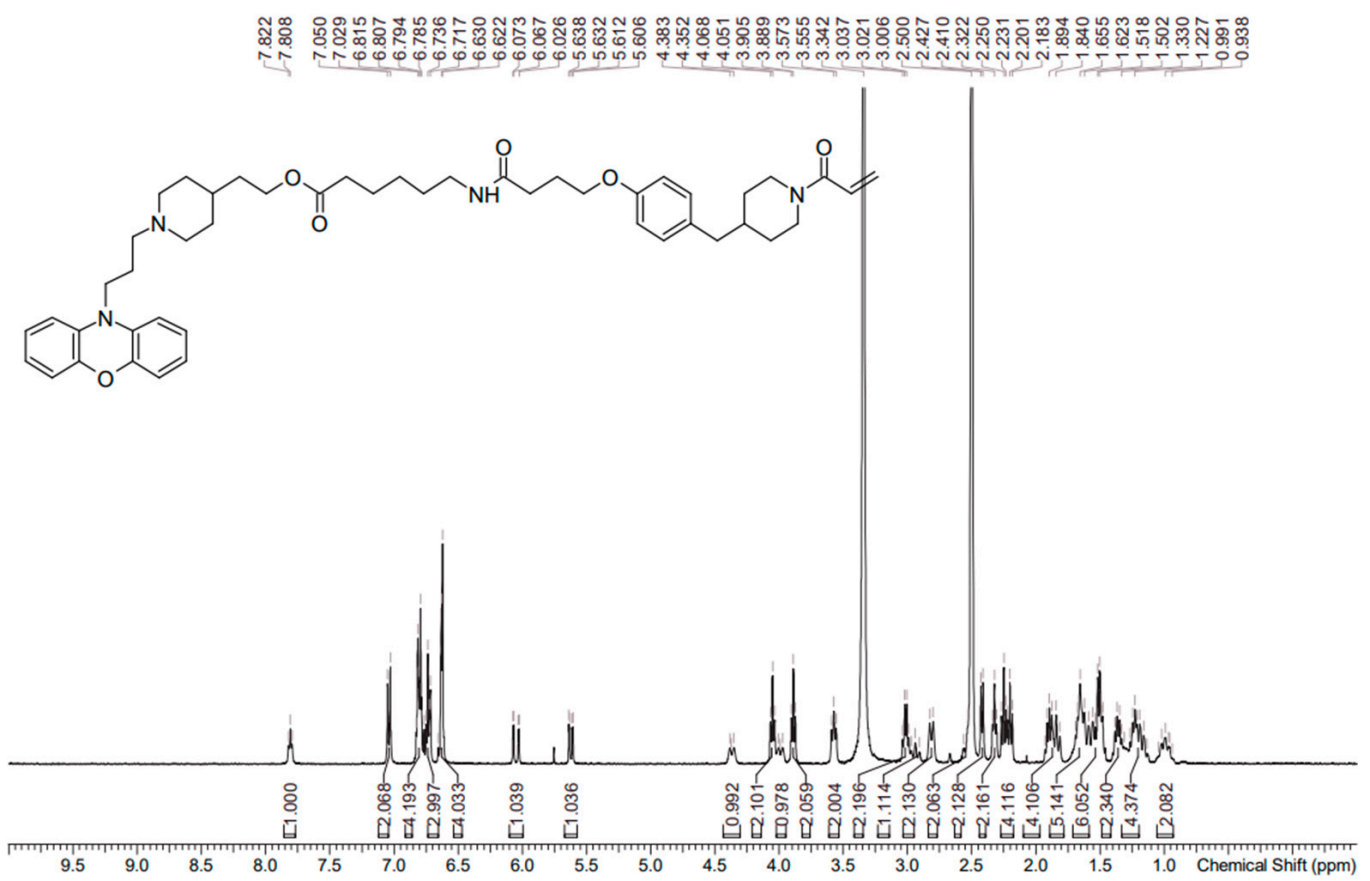


#### 2.3.2. Synthesis of Compound **4**

Compound **4** was prepared following the workflow shown below. 4-(4-Methoxybenzyl)piperidine hydrochloride, **5**, was deprotected with boron tribromide. The piperidine nitrogen in **6** was then protected as a *t*-Boc carbamate, and **7** was treated with methyl 4-bromobutanoate to generate the ester **8**. Mild saponification gave **9,** which was used to make amide **10** via cross-coupling with 2-(7-azobenzotriazole)-*N*,*N*,*N′*,*N′*-tetramethyluronium hexafluorophosphate (HATU). After acidic deprotection, **4** was obtained from **11** and acryloyl chloride.


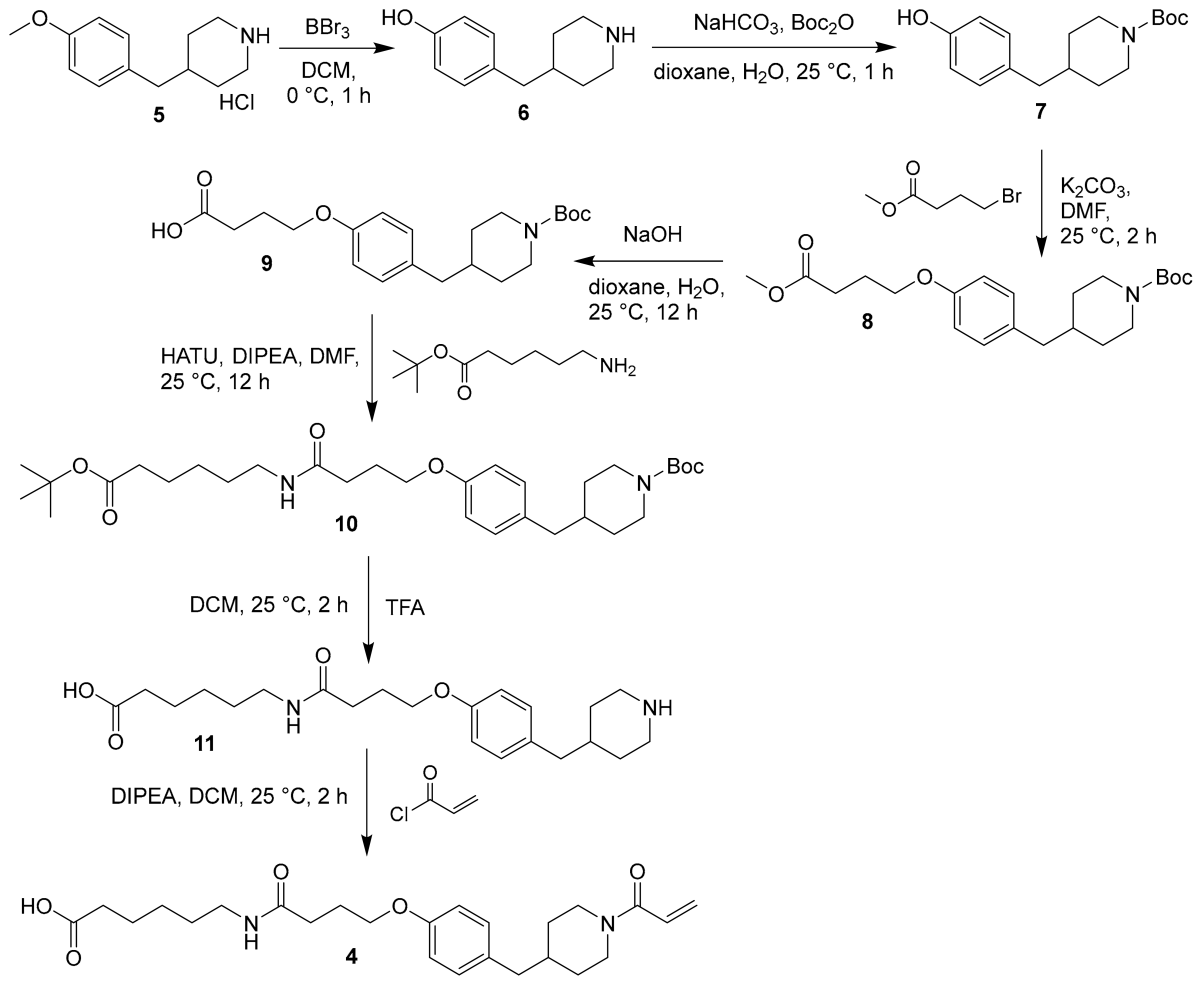


### 2.4. Cell Culture

Mammalian cell lines RH28, RD (ATCC catalog no. CCL-136, RRID:CVCL_1649, Manassas, VA, USA), and HS27 (ATCC catalog no. CRL-1634, RRID:CVCL_0335, Manassas, VA, USA) were maintained in high-glucose Dulbecco’s Modified Eagle Medium (DMEM) (Gibco #11965118, Grand Island, NY, USA) supplemented with 10% fetal bovine serum (FBS; Sigma #F0926, St. Louis, MO, USA). RH30 (ATCC catalog no. CRL-2061, RRID:CVCL_0041, Manassas, VA, USA), RH41 (RRID:CVCL_2176), RH5, RH4^PF-FKBP^, and TC-71 (RRID:CVCL_2213) cells were grown in RPMI 1640 medium (Gibco #11875119, Grand Island, NY, USA) supplemented with 10% FBS (Sigma #F0926, St. Louis, MO, USA). All cell lines were grown at 37 °C in a high-humidity 5% CO_2_ atmosphere incubator. To maintain high-quality performance in the experiments, cell lines were not kept in culture after passage 25, except HS27 until passage 33, and were continuously checked for mycoplasma contamination with the latest test on 6 May 2026, using the MycoAlert system according to manufacturer’s instructions (Lonza catalog no. LT07-318, Portsmouth, NH, USA).

RH41 cell lines were kindly provided by Dr. Peter Houghton, University of Texas Health San Antonio (San Antonio, TX, USA), as well as RH28 and RH5, which were kindly provided by Dr. Frederic G. Barr, National Cancer Institute (Bethesda, MD, USA). TC-71 cells were kindly provided by Dr. Tim Triche, Children’s Hospital Los Angeles (Los Angeles, CA, USA).

2x*HA-FKBP12^F36V^* degron tag knock-in cells RH4 were kindly provided by Dr. Stengel, Albert Einstein College of Medicine (New York, NY, USA) [39].

### 2.5. Immunoblotting

Cells were lysed on ice for 30 min in phospho-lysis buffer (PLB; HEPES pH 7.9 50 mM, NaCl 100 mM, NaPP 4 mM, EDTA 10 mM, NaF 10 mM, Triton 1%) containing protease and phosphatase inhibitors (Vanadate 2 mM, PMSF 1 mM, Aprotonin 2 µg/mL, and Leupeptin 2 µg/mL) and vortexed. After centrifugation for 15 min at 20,000× *g* at 4 °C, protein concentration of the lysates was assessed via Pierce BCA assay (Thermo Scientific catalog no.: #A65453, Waltham, MA, USA) and denatured by heating at 95 °C for 5 min in Laemmli sample buffer (5X stock solution containing 62.5 mM Tris-HCl (pH 6.8, 4% SDS), 20% glycerol, 2% SDS, and 5% β-mercaptoethanol, supplemented with a trace amount of bromophenol blue as a tracking dye). Proteins were separated by SDS-PAGE and transferred to an Immobilon-P PVDF membrane (Sigma catalog no.: #IPVH00010, St. Louis, MO, USA) via overnight transfer at 4 °C. Membranes were then blocked in 5% nonfat dry milk in TTBS (Tween-Tris-buffered saline; 20 mmol/L Tris-HCl, pH 7.5, 150 mmol/L NaCl, 0.5% Tween 20) for 1 h followed by primary antibody addition in 5% BSA in TTBS over night at 4 °C. Immunoblotting was performed using anti-FOXO1 (1:2000, Cell Signaling Technology catalog no. 2880, RRID:AB_2106495, Danvers, MA, USA), MYOD1 (1:1000, Cell Signaling Technology catalog no. 13812, RRID:AB_2798320, Danvers, MA, USA), MYOG (1:1000, Novus catalog no. NB100-56510, RRID:AB_838604, Centennial, CO, USA), myosin heavy chain (1:1000, R&D systems catalog no. MAB4470, RRID: AB_1293549, Minneapolis, MN, USA), ubiquitin (1:1000, Cell Signaling Technology catalog no. 3936, RRID:AB_331292, Danvers, MA, USA), SKP1 (1:1000, Cell Signaling Technology catalog no. 2156, RRID:AB_2270271, Danvers, MA, USA), PAX3 (1:500, Cell Signaling Technology catalog no. 12412, RRID:AB_2636922, Danvers, MA, USA), FLI1 [40], and actin-HRP (1:25,000, Abcam catalog no. ab20272, RRID:AB_445482, Waltham, MA, USA) antibodies. The membrane was then washed three times in TTBS, incubated in secondary antibody (anti-rabbit or -mouse) coupled to horseradish peroxidase (Thermo Fisher Scientific catalog no. NA934; catalog no. NA9311ML, Waltham, MA, USA) in 5% nonfat dry milk in TTBS for 1 h, and subsequently washed three times with TTBS. The blots were developed using Millipore Immobilon Western chemiluminescent horseradish peroxidase substrate per the manufacturer’s instructions (Millipore catalog no. WBKLS0500, Burlington, BA, USA). Chemiluminescence was detected using a LI-COR Odyssey Fc imaging system (RRID:SCR_023227). Bands were quantified using Fiji Software (ImageJ 1.54p (2.16.0; Java 1.8.0_322)) and normalized to actin as a loading control.

### 2.6. Immunoprecipitation

For immunoprecipitation, protein lysates were prepared in PLB as described above, followed by rotating lysates overnight at 4 °C containing rabbit IgG antibody control (Cell Signaling Technology #2729, RRID:AB_1031062, Danvers, MA, USA) or anti-FOXO1 antibody (1:2000, Cell Signaling Technology catalog no. 2880, RRID:AB_2106495, Danvers, MA, USA) at 1:140 dilution. The next day, 25 µL protein G magnetic beads (Active Motif #53033, Carlsbad, CA, USA) were added, and samples were rotated for 90 min at 4 °C. Samples were washed three times in PLB and finally eluted by boiling at 95 °C for 5 min in sample buffer.

### 2.7. Immunofluorescence Imaging

RH41 and RD cells were seeded 20,000 cells/well in 6-well plates containing sterilized coverslips and treated for 7 days with redosing of drugs after 3 days. Cells were then fixed in 3.7% paraformaldehyde for 15 min and permeabilized with 0.5% Triton X-100 in PBS for 15 min. After blocking cells for 1 h in blocking solution, 5% BSA in PBS containing 0.05% Tween-20 (PBST), cells were incubated for 30 min in Image-iT™ FX Signal Enhancer (Thermo Fisher Scientific Cat. No.: I36933, Waltham, MA, USA). Next, cells were incubated in 1:50 myosin heavy chain (1:1000, R&D systems catalog no. MAB4470, RRID: AB_1293549, Minneapolis, MN, USA) primary antibody in 0.5% BSA in PBST for 1 h followed by co-staining of a 1:500 dilution of goat α-mouse IgG Alexa Fluor 488 secondary antibody (Invitrogen, Carlsbad, CA, USA, #A11001) in 0.5% BSA in PBST and 0.25× dilution of Texas Red-X Phalloidin (Thermo Fisher Scientific, #T7471, Waltham, MA, USA) for 1 h. The cells were finally counterstained with 300 nM 4′,6-diamidino-2-phenylindole (DAPI) for 10 min and mounted on glass slides with ProLong Diamond Antifade Mountant (Thermo Fisher Scientific, #P36965, Waltham, MA, USA). All steps were performed at room temperature. Microscopy images were acquired using a Leica SP8 confocal microscope (Wetzlar, Germany) and analyzed by its LAS X v3.5.7 software. Quantification of images was performed using CellProfiler software version 4.2.8 (RRID:SCR_007358).

### 2.8. PAX3::FOXO1 Purification

A recombinant PAX3::FOXO1 protein was purified as described previously [31]. Briefly, full-length PAX3::FOXO1 (GenBank accession code: AAC50053.1) containing a carboxy-terminal 12 His tag, an amino-terminal BioEase tag, and an internal HA tag close to the amino-terminal in the pET104.1 DEST plasmid was used for expressing the protein in *Escherichia coli* strain BL-21 (DE3). A single bacterial colony from an agar plate was grown in 2.5% LB liquid medium with 100 ug/mL ampicillin at 37 C in a shaker (250 rpm) overnight. The bacteria were then transferred to 1 L fresh LB medium without ampicillin at 1:200 dilution and grown in a shaker at 37 C until the OD at 600 nm reached 0.6. Isopropyl-β-D-thiogalactopyranoside (1 mM) was added to induce recombinant protein expression, and the sample was grown for another 4 h. Bacterial pellets were prepared by centrifugation and stored at −80 until purification. On the day of purification, bacterial pellets were thawed on ice and dissolved in 10 mL A1 buffer (20 mM Na-P buffer, pH 7.4, 500 mM NaCl, 30 mM imidazole) plus EDTA-free protease inhibitor tablet (MilliporeSigma catalog no: 11836170001, Burlington, MA, USA). The sample was incubated on ice for 15 min with 0.2 mg/mL lysozyme and then sonicated five times with 15 s bursts on ice. The sonicated sample was centrifuged (13,500× *g*) for 10 min at 4 C and then filtered through a 0.45 uM microfilter to remove any aggregates. A HisTrapHP 1 mL column was used for purification in AKTA Pure 25 Explorer (Cytiva, Marlborough, MA, USA), where the column was washed with water, followed by A1 buffer to equilibrate the column before sample application and consequent wash. Recombinant protein was eluted from the column with a linear gradient to B1 buffer (20 mM Na-P buffer, pH 7.4, 500 mM NaCl, 1 M imidazole).

### 2.9. Surface Plasmon Resonance

All experiments were conducted using a Biacore T200 instrument (Marlborough, MA, USA) with a CM5 chip at 25 °C. Recombinant PAX3::FOXO1 and FLI1 DNA-binding domain (DBD) were immobilized as ligands onto the CM5 sensor surface, using standard amine coupling chemistry. PAX3::FOXO1 was diluted in 10 mM sodium acetate buffer at pH 5.0 and immobilized onto a flow cell (FC) to levels of ~5000–5400 RU. FLI1 DBD was diluted in 10 mM sodium acetate buffer at pH 4.0 and immobilized onto another FC to levels of ~800–1450 RU. A neighboring FC with no proteins captured on it was used as the reference (RFC) for both FCs with recombinant proteins (Active FCs, AFCs). The RFC was modified using the same surface chemistry as AFCs. PBS-P (20 mM phosphate buffer pH 7.5, 137 mM NaCl, 2.7 mM KCl, 0.05% *v*/*v* surfactant P20) (Cytiva, Catalog no. 28995084, Marlborough, MA, USA) was used as the immobilization running buffer. Small molecules (SMs) in 10 mM DMSO stocks were used as analytes to flow over the ligand-immobilized surfaces. SM solutions were prepared in PBS-P supplemented with 5%DMSO *v*/*v* and injected at a flow rate of 50 μL/min to RFC and AFCs. PBS-P supplemented with 5% DMSO *v*/*v* buffer was used as the running buffer during SM-protein binding. One 20 s pulse of 2 M NaCl was injected for surface regeneration. Injected SM (analyte) concentrations were from 40 μM to 1.25 μM (two-fold dilutions). Each concentration of all analytes was injected in duplicate for technical reproducibility. Sensorgrams obtained for analyses were both blank (buffer only) and reference (sensorgrams corresponding to the RFC) subtracted. The SM binding sensorgrams were evaluated by fitting to a steady-state affinity or 1:1 kinetics model. SPR results are concluded based on two sets of SM-protein binding data for each SM.

### 2.10. Cell Viability and Drug Synergy Scoring

1800 cells/well were seeded in 384-well plates in 30 µL media. Subsequently, test compounds were added in technical triplicate in a concentration series followed by cell viability measurement using the CellTiter-Blue assay and determining fluorescence following manufacturer’s instructions (Promega catalog no. G808A, Madison, WI, USA) on a BioTek Synergy H4 plate reader (Winooski, VT, USA, RRID:SCR_019750). Mean values +/− standard deviations (SD) were determined, and IC50 values calculated from dose-response curves by GraphPad Prism (version 10, log(inhibitor) versus normalized response, variable slope).

For drug synergy scores, concentrations of vincristine were combined with a concentration series of PROTAC, and cell viability was determined using CellTiter-Blue. Cell viability was normalized to the DMSO control and analyzed via SynergyFinder 3.0 [41] using the four-parametric logistic regression curve-fitting algorithm, and synergy scores were calculated with the Bliss model. Antagonistic interactions of the tested drugs are described by scores less than −10, scores between −10 and 10 suggest additivity of the tested drugs, and scores larger than 10 suggest synergy between the tested drugs.

### 2.11. Colony Formation Assay

Cells were seeded in 12-well plates at 500 cells/well (TC71), 1000 cells/well (RD), or 2000 cells/well (RH41, RH4, HS27) in 1 mL media. Tested compounds were added with DMSO content normalized over all wells (never exceeding 1%). Cells were treated for 7d (TC71) or 10d (RD, RH41, RH4, HS27), washed with PBS, and fixed in 10% Neutral Buffered Formalin (10%)(Fisher Scientific, Catalog no. 22-110-869, Waltham, MA, USA) for 20 min. After washing with ddH20, 0.5% crystal violet solution (10% methanol) was added to the cells for 20 min, followed by washing twice with water. After wells were dried, images were taken using a GelCount colony counter (Oxford Optronix Ltd., Cambridge, UK, RRID:SCR_023219). For quantification, 10% acetic acid (Sigma-Aldrich, Catalog no. 695092, St. Louis, MO, USA) was added to each well for destaining, and plates were left shaking on a Rocking Shaker for 20 min. Absorbance of the extracted crystal violet was measured at 590 nm for each well in triplicate in a 96-well plate with a BioTek Synergy H4 plate reader (Winooski, VT, USA, RRID:SCR_019750).

### 2.12. Cell Cycle Analysis

300,000 RH41 or RD cells were seeded per well in a 6-well plate and treated for 24 h with DMSO, 5 µM PFP-16 or PFP-4. Cells were then harvested and fixed in ethanol on ice for 20 min, followed by propidium iodide staining (Sigma-Aldrich Catalog no. P4170, St. Louis, MO, USA) for 30 min at room temperature and 30 min at 4 °C protected from light. Flow cytometry data acquisition was performed using a BD SORP Fortessa instrument (Franklin Lakes, NJ, USA), and analysis was performed with FCS Express 7 (v7.28.0035).

### 2.13. Soft Agar Assay

For the bottom agar layer, 0.6% agar in cell culture media was prepared, and 0.5 mL of this mixture was distributed to each well in a 12-well plate. After solidification of the bottom layer, 1000 (TC71), 5000 (RH41), or 10 000 (RH4) cells per well were seeded in 1 mL of a 0.4% agar top layer containing DMSO or PROTACs. Once the top layer containing cells and drugs solidified, the plates were placed in the 37 °C incubator and grown for 10d (TC71) or 21d (RH41, RH4). Drugs were re-dosed every 4–5 days in 150 µL media on top of each well. Images of the plates were taken using a GelCount colony counter (Oxford Optronix Ltd., Cambridge, UK, RRID:SCR_023219) and quantified using the accompanying software (GelCount version 1.4.2.0).

### 2.14. RNA Isolation, Reverse Transcription, and qRT-PCR

For RNA isolation, the RNeasy Plus mini kit (Qiagen #74134, Hilden, Germany) was used following the manufacturer’s instructions. RNA quantity and quality were determined via NanoDrop (Thermo Fisher Scientific, Waltham, MA, USA), and 1 µg of RNA was further used for reverse transcription and cDNA synthesis performed via SuperScript VILO cDNA synthesis Kit (Thermo Fisher Scientific, catalog no. 11754050, Waltham, MA, USA) utilizing the oligo(dT) primers. Generated cDNA was used for qPCR utilizing SYBR Green Readymix (Sigma #KCQS00, St. Louis, MO, USA) according to the manufacturer’s instructions and run on a Roche Lightcycler 480 instrument (Mannheim, Germany) with the following conditions: 95 °C for 10 min, [95 °C for 15 s, 59 °C for 30 s, and 72 °C for 30 s] × 40, followed by melting curve determination. Differences in gene expression were calculated using the ∆∆C(T) method, where 18S functioned as a housekeeping gene to which the relative mRNA amounts were normalized. The primers listed in Table 1 were used for the experiments with a final concentration of 250 nM in each reaction. Statistical analysis was assessed in GraphPad PRISM (RRID: SCR_002798), where values were normalized to the DMSO control condition as expressing genes at 100%, and these normalized values were assessed by an Ordinary One-way ANOVA.

### 2.15. RNA-Sequencing

RNA was collected from RH41 cells treated with 5 µM PFP-16 or DMSO control in three biological replicates for 7 days. RNA was extracted using the Qiagen RNeasy kit, according to the manufacturer’s instructions. cDNA library preparation and RNA sequencing were performed by NovoGene (Sacramento, CA, USA) using poly A enrichment and NovaSeq X Plus Series (PE150) for Illumina (San Diego, CA, USA) paired-end sequencing (150 bp reads). Reads were aligned to the human genome (GRCh38.p13) using HISAT2 (RRID:SCR_015530), followed by counting raw reads via featureCounts (RRID:SCR_012919) in Galaxy (GVL 4.4.0) [42]. Downstream analyses were performed in R using packages DESeq2 (R version 4.5.3) (RRID:SCR_015687) and EdgeR (RRID:SCR_012802). Genes with counts-per-million values less than 1 in at least 2 samples were filtered out. Gene Set Enrichment Analysis (GSEA, RRID:SCR_003199) was performed on differentially expressed genes to identify enriched gene sets in PFP-16-treated cells as specified in figure legends.

### 2.16. Plasmid Transfection

RH28 and TC71 cells were transfected with PAX3::FOXO1, which was kindly provided by Dr. Frederic G. Barr, National Cancer Institute (Bethesda, MD, USA), FLAG-FOXO1A (Addgene, Watertown, MA, USA, catalog no. 153141), or FLAG-PAX3 (Addgene, Watertown, MA, USA, catalog no. 78335) plasmids using X-tremeGENE 9 DNA transfection reagent (Sigma #6365787001, St. Louis, MO, USA) according to the manufacturer’s instructions. 6–8 h post-transfection, cells were treated for 24 h with PFP-16.

### 2.17. Statistical Analysis

Data are presented as mean  ±  SD. Statistical analyses were performed using the GraphPad Prism (version 10) software or R (R version 4.5.3). Appropriate statistical analyses are described in figure legends and data are visualized using the listed symbols: ns: not significant, *p*-value > 0.05, * *p*-value: 0.01–0.05, ** *p*-value: 0.001–0.01, *** *p*-value: >0.001, **** *p*-value: <0.0001.

## 3. Results

### 3.1. Discovery of PAX3::FOXO1-Targeting PROTACs

**Figure 1:**
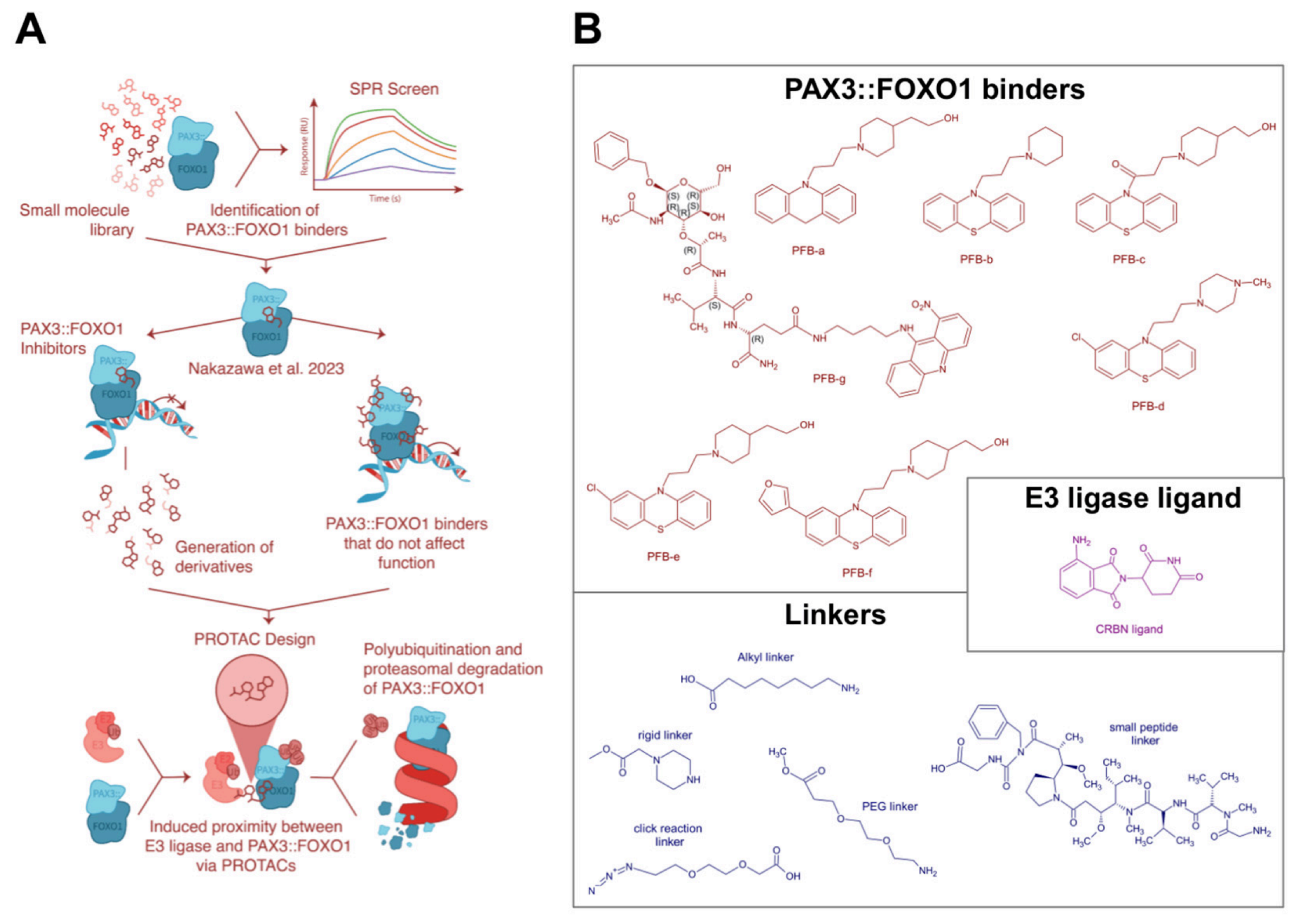
Design of first-generation PAX3::FOXO1-targeting PROTACs. (**A**) Schematic overview of the design of first-generation CRBN-based PAX3::FOXO1-targeting PROTACs. (**B**) Chemical structures of PROTAC components depicted. PROTACs consist of different PAX3::FOXO1 binders attached to the CRBN E3 ligase ligand via various linkers [31].

**Figure 2:**
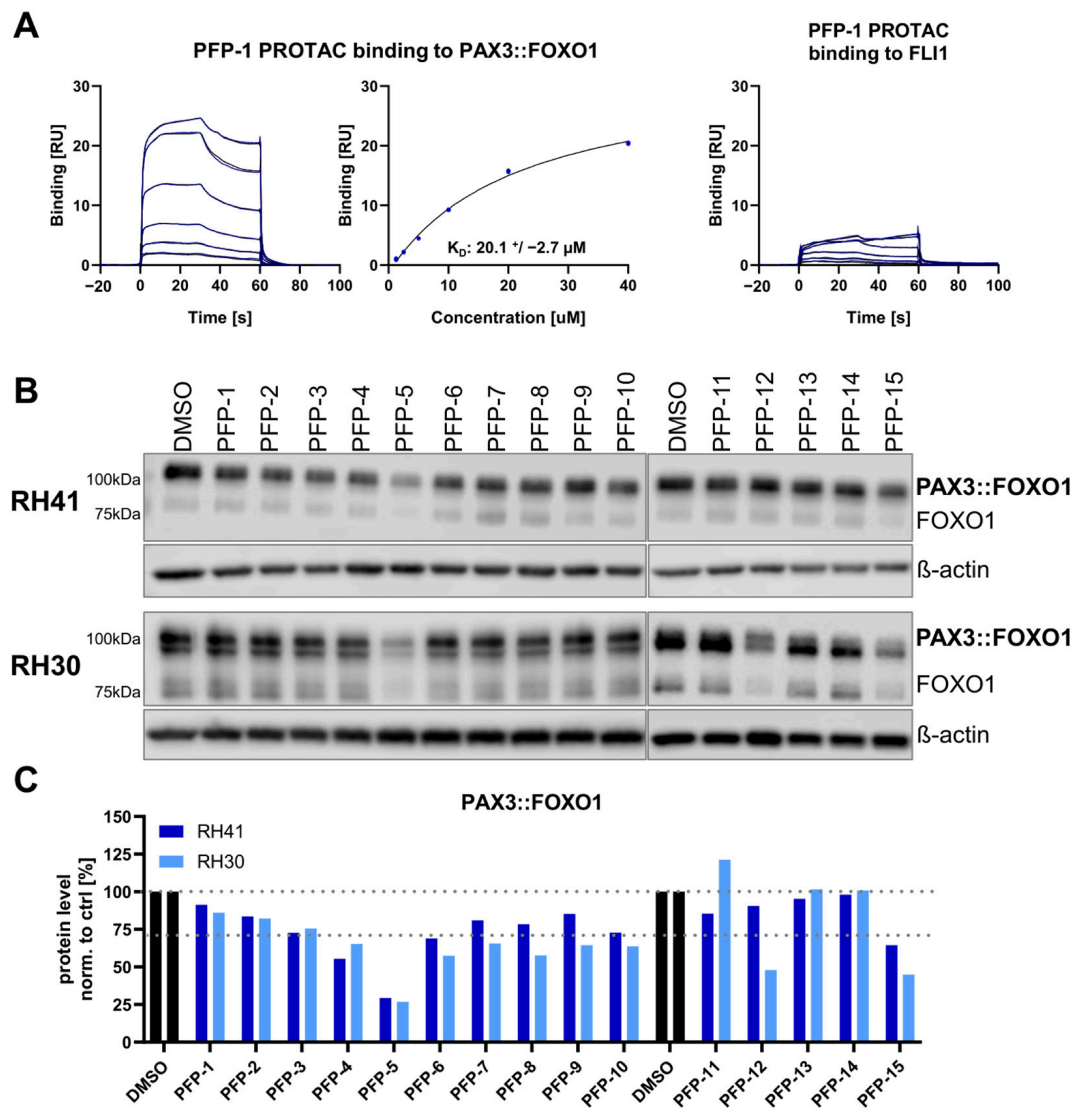
CRBN-based PAX3::FOXO1-PROTACs directly bind to PAX3::FOXO1 protein and degrade it in FP-RMS cells. (**A**) SPR sensorgram for the PROTAC **PFP-1** demonstrates direct binding to PAX3::FOXO1 protein. FLI1 DNA-binding domain was used as the negative control. Proteins were immobilized on a CM5 chip, and the PROTAC was used as the analyte at 6 different concentrations (40 µM, 20 µM, 10 µM, 5 µM, 2.5 µM, and 1.25 µM). K_D_ value was determined via steady-state affinity fitting and represents the mean and standard deviation (mean ± s.d.) from three independent experiments. Symbols in the binding vs. concentration plot correspond to the response values towards the end of association signals (60 s) from the SPR data (binding vs. time graph), and the continuous line represents the curve fit used for K_D_ calculation. (**B**) RH41 and RH30 cells were treated for 24 h with 5 µM or 10 µM of indicated PROTACs, respectively. (**C**) Quantification of PAX3::FOXO1 western blots from panel (**B**) shown as a bar graph. Bands were first normalized to actin and then to the DMSO control.

We screened small molecule libraries for compounds that can directly bind to PAX3::FOXO1 protein using SPR in a previously published study [31]. We identified 119 small molecules as primary hits that can directly interact with the recombinant PAX3::FOXO1 protein. When the initial hits were evaluated for inhibiting PAX3::FOXO1 function in FP-RMS cells, piperacetazine emerged as the lead molecule, which prompted us to synthesize derivatives of piperacetazine (Figure 1A). From this arsenal of PAX3::FOXO1 binding molecules, we selected both the compounds that directly bound to PAX3::FOXO1 and inhibited its function as well as the compounds that directly bound to the protein without affecting its function (Table S1). We initially designed 15 first-generation **P**AX3::**F**OXO1-targeting **P**ROTACs (**PFP-1** to **-15**) by selecting seven different **P**AX3::**F**OXO1 **b**inder small molecules (**PFB-a** to **-g**) to be conjugated through five different linkers to the CRBN E3 ligase ligand thalidomide (Figure 1B, Tables S1 and S2). Linker types were chosen from published commonly used PROTAC designs [43,44,45], including linkers used in Protac-1 (alkyl) [32], dBET1 (alkyl) [46], ARV-110 and ARV-471 (vepdegestrant, FDA-approved) (rigid) [47,48], and MZ1 (PEG) [49]. We first confirmed that the PROTACs retained the ability to directly bind to PAX3::FOXO1 protein via SPR (Figure 2A and Figure S1A–D). Recombinant purified PAX3::FOXO1 was immobilized on a CM5 Biacore sensor chip to which **PFP-1** bound with a K_D_ value of 20.1 ± 2.7 µM (Figure 2A). Although **PFP-1** showed weak binding close to the baseline signal towards the FLI1 DNA-binding domain (DBD), used here as a negative control, the interaction did not follow a dose-response relationship, did not fit the 1:1 binding model for determination of a K_D_ value, and therefore it was considered non-specific (Figure 2A).

We tested the first-generation PFPs for their ability to degrade endogenous PAX3::FOXO1 protein in FP-RMS cell lines RH41 and RH30. We observed various degrees of PAX3::FOXO1 degradation, with some PROTACs decreasing PAX3::FOXO1 protein levels to more than 50% (Figure 2B,C). While **PFP-5** and **PFP-15** decreased PAX3::FOXO1 protein levels effectively in both cell lines, they also induced non-specific cell death of all tested cell lines regardless of fusion protein status (Figure S2A), which suggested that the decrease in PAX3::FOXO1 protein levels may have been due to cells dying at the tested dose. Further, **PFP-5** treatment decreased *PAX3::FOXO1* mRNA levels (Figure S2B). These observations suggested a nonspecific activity of **PFP-5** and **PFP-15**. Additionally, their large molecular weights exceeding 1400 Da (Table S2) were also considered unfavorable, and, therefore, **PFP-5** and **PFP-15** were not evaluated further in any other follow-up experiments. **PFP-3, PFP-4, PFP-6**, and **PFP-10** reduced PAX3::FOXO1 protein levels in both cell lines by 30% or more without decreasing *PAX3::FOXO1* mRNA levels, and without inducing acute cell death, indicating that the drugs are acting on the protein as expected (Figure 2B,C and Figure S2A,B).

### 3.2. SKP1 E3 Ligase Is Highly Expressed and a Dependency in FP-RMS and Improves PROTAC Efficacy

**Figure 3:**
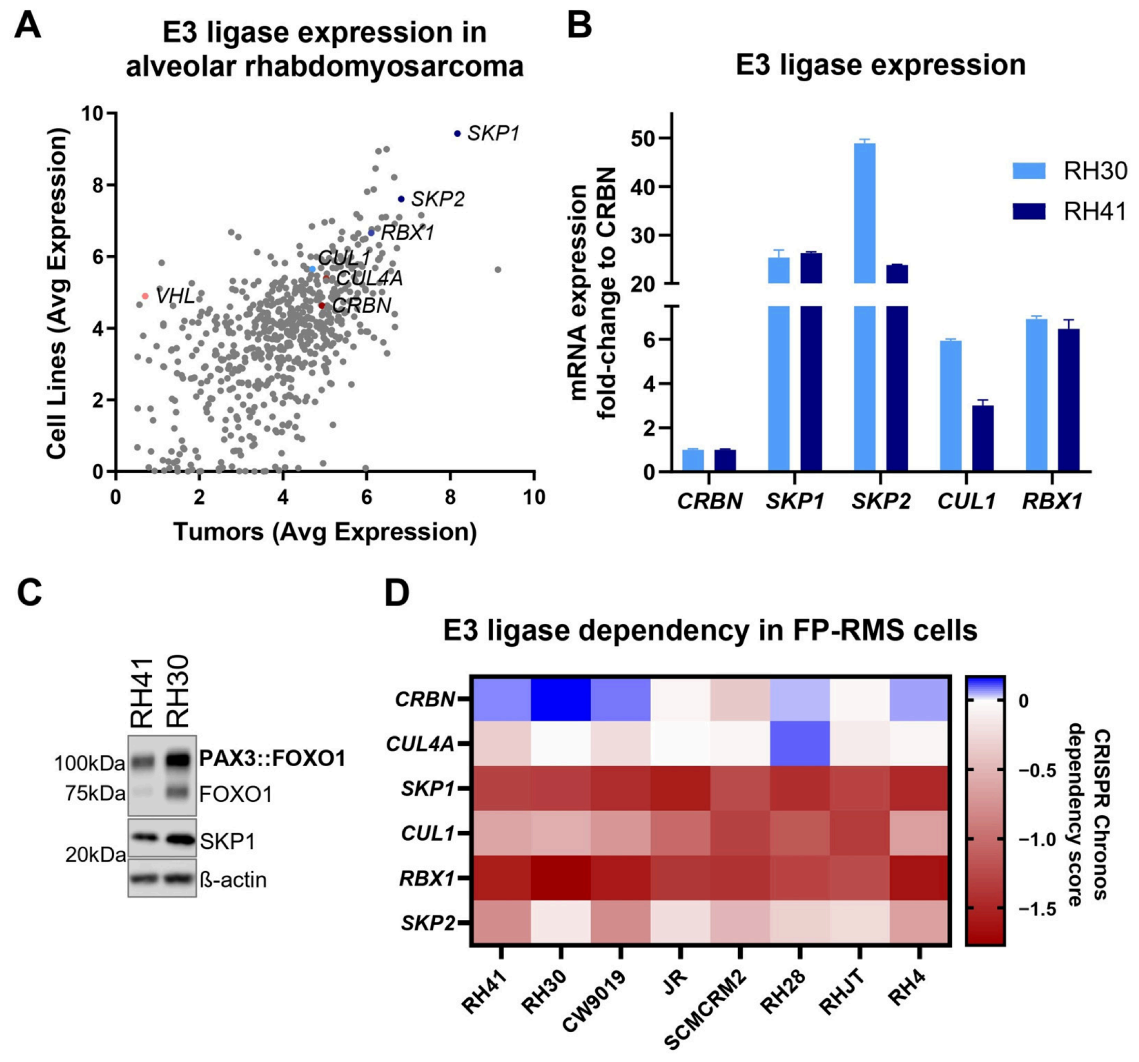
E3 ligase *SKP1* is highly expressed and dependent in FP-RMS cells. (**A**) E3 ligase mRNA expression across FP-RMS cell lines and patient tumors. E3 ligase list derived from Liu et al. [58] and mRNA expression data accessed for FP-RMS cell lines from DepMap and alveolar RMS tumors from Davicioni et al. (GEO ID: GSE92689) [64]. (**B**) qRT-PCR of E3 ligase mRNA expression in RH41 and RH30 cells. Normalized to *18S* gene expression as a control and fold-change to *CRBN* is displayed for each cell line. (**C**) SKP1 protein expression was evaluated by western blot in RH41 and RH30 cell lysates. (**D**) Dependency scores for selected E3 ligases across FP-RMS cell lines derived from depmap.org. A smaller CRISPR Chronos score represents higher dependencies.

**Figure 4:**
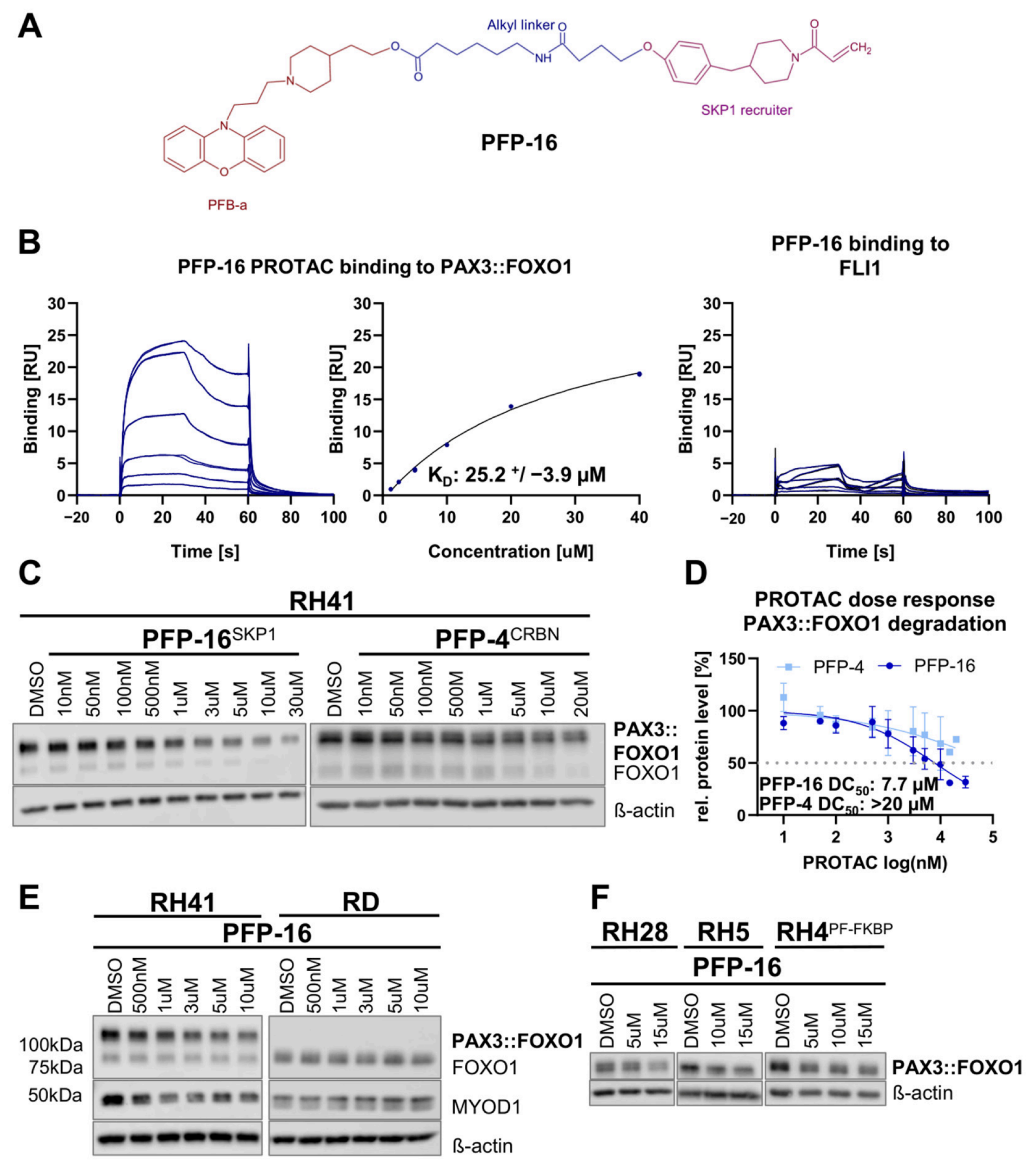
SKP1-based PROTAC directly binds to PAX3::FOXO1 protein and degrades it in FP-RMS cells. (**A**) Chemical structure of SKP1-based PROTAC **PFP-16**. (**B**) SPR sensorgram for **PFP-16** demonstrates direct binding to the PAX3::FOXO1 protein. The FLI1 DNA-binding domain was used as the negative control. Proteins were immobilized on a CM5 chip, and the PROTAC was used as the analyte at six different concentrations (40 µM, 20 µM, 10 µM, 5 µM, 2.5 µM, and 1.25 µM). K_D_ value was determined via steady-state affinity fitting and represented as the mean and standard deviation (mean ± s.d.) from three independent experiments. Symbols in the binding vs. concentration plot correspond to the response values towards the end of association signals (60 s) from the SPR data (binding vs. time graph), and the continuous line represents the curve fit used for K_D_ calculation. (**C**) Western blot staining for the PAX3::FOXO1 protein following 24 h treatment of SKP1-based **PFP-16** and CRBN-based **PFP-4** in RH41 cells. (**D**) Protein quantification from images in panel C and two other replicate experiments (n = 3) was used to calculate half-maximal degradation concentration (DC_50_). The PAX3::FOXO1 signal was first normalized to the actin level and then expressed as % of DMSO control. (**E**) Western blot staining for PAX3::FOXO1 and MYOD1 with actin as loading control following 24 h **PFP-16** treatment in FP-RMS RH41 and FN-RMS RD cells. A representative image from three independently performed experiments is shown. (**F**) Western blot staining for PAX3::FOXO1 and actin following 24 h **PFP-16** treatment in RH28, RH5, and RH4^PF-FKBP^ cells. A representative image from two independently performed experiments is shown.

We further optimized the PFPs by evaluating different E3 ligases other than CRBN that might achieve better degradation of PAX3::FOXO1. The human genome encodes more than 600 E3 ligases, which mediate specificity within the ubiquitin-proteasome system (UPS); however, only a few have been incorporated into PROTAC designs [50,51,52,53,54,55,56,57]. To select an E3 ligase that may be more efficient than CRBN in FP-RMS cells, we first investigated E3 ligase expression levels in FP-RMS cell lines and patient tumor samples. A comprehensive list of E3 ligases and related proteins (such as E3 ligase receptors) was derived from Liu et al. [58] and used to mine publicly available RMS gene expression datasets. FP-RMS cell line expression data were accessed via DepMap (depmap.org), and RMS tumor data were derived from Davicioni et al. (GEO ID: GSE92689) [59]. *SKP1* stood out as highly expressed in both established FP-RMS cell lines and patient tumor samples (Figure 3A). We validated that *SKP1* and related complex members (*SKP2, CUL1, RBX1*) are expressed at higher levels than *CRBN* in FP-RMS cells via qRT-PCR (Figure 3B) and confirmed that the SKP1 protein was expressed in two FP-RMS cell lines by western blot (Figure 3C). In addition to E3 ligase expression, we also used dependency data to guide our E3 selection process for the new PROTAC designs. Similar to all targeted therapy approaches, PROTACs are prone to acquired resistance mechanisms in cancer via mutations at the E3 ligase loci or loss of target E3 ligase expression [33,60,61]. Relying on non-essential E3 ligases such as CRBN represents a particular risk, as cancer cells could quickly evade PROTACs through loss of the non-essential E3 ligase function without impairing their viability [33,62]. By utilizing the dependency data from DepMap, we observed that, unlike *CRBN* or complex member *CUL4A*, *SKP1* and complex members have strong dependencies in FP-RMS cell lines (Figure 3D), thus being less prone to resistance mechanisms when recruited by PROTACs. A functional SKP1 recruiter was recently described and used for designing PROTACs targeting BRD4 and AR [63]. Accordingly, we designed the second generation of PFP by replacing the CRBN binding unit with an SKP1 binding molecule. We used the binder that showed robust PAX3::FOXO1 degradation in the first generation PFPs, namely **PFB-a** (incorporated in PROTACs **PFP-1-5**), and the alkyl linker that was used for **PFP-2, PFP-6-10**, and **PFP-12** to generate the second generation SKP1-based PROTAC **PFP-16** (Figure 4A). PFP-16 retained direct binding capability to PAX3::FOXO1 protein with a binding affinity of K_D_ = 25.2 +/− 3.9 µM as determined via SPR (Figure 4B).

We directly compared the degradation efficacy of the SKP1-based **PFP-16** compound to **PFP-4**, the CRBN-based counterpart with the same PAX3::FOXO1 binder, which worked best in our initial screening (Figure 2B). We observed that the SKP1-based PROTAC more potently degraded endogenous PAX3::FOXO1 protein with a half maximal degradation concentration (DC_50_) of 7.7 µM in RH41 cells compared to the CRBN-based **PFP-4**, which did not achieve degradation greater than 50% at the highest tolerated dose of 20 µM (Figure 4C,D). Moreover, the concentration-dependent degradation of PAX3::FOXO1 protein via **PFP-16** treatment in FP-RMS RH41 cells led to a decrease in MYOD1, a canonical PAX3::FOXO1 target gene (Figure 4E). In RD cells, an FN-RMS cell line, we did not observe a decrease in FOXO1 or MYOD1 levels following **PFP-16** treatment, supporting on-target effects of **PFP-16** (Figure 4E). Moreover, **PFP-16** was able to degrade PAX3::FOXO1 protein in multiple FP-RMS cell lines (Figure 4F), overall leading us to pursue **PFP-16** as a potent PROTAC against PAX3::FOXO1 for further downstream analysis. We used RH4 cells with endogenous PAX3::FOXO1-2xHA-FKBP^12F36V^ degron tag [39], hereafter referred to as RH4^PF-FKBP^, as an additional FP-RMS cell line to characterize **PFP-16** activity. RH4^PF-FKBP^ cells were created by Dr. Stengel through a CRISPR-based genome editing approach to integrate a 2xHA-FKBP12F36V degron tag (FKBP) into the last exon of the endogenous PAX3::FOXO1 gene. In this model, FKBP-tagged PAX3::FOXO1 becomes quickly degraded following dTAG-47 treatment. Hence, RH4^PF-FKBP^ served as a positive control for specific and selective PAX3::FOXO1 degradation (Figure S3A) for downstream analyses of **PFP-16**, since dTAG-47 selectively degrades FKBP^12F36V^ degron-tagged proteins [46,65,66].

Interestingly, **PFP-16** activity was dependent on the duration of the treatment, as we observed little PAX3::FOXO1 degradation at 2 h, 50% degradation of PAX3::FOXO1 protein following 24 h treatment, and >70% degradation after a 7-day treatment (Figure S3B,C). At the same time, we tracked *PAX3::FOXO1* mRNA levels to exclude any indirect effects of PROTAC treatment on PAX3::FOXO1 protein levels. *PAX3::FOXO1* mRNA levels fluctuated over time; however, they remained stable even when a dramatic PAX3::FOXO1 protein decrease was observed at 7 days (Figure S3C). Consistent with the 24 h treatment (Figure 4C), the SKP1-based PROTAC **PFP-16** more effectively degraded PAX3::FOXO1 protein after 7 days compared to CRBN-based PROTACs **PFP-3** and **PFP-4** (Figure S3D).

While we observed effective PAX3::FOXO1 degradation in RH4^PF-FKBP^ cells with **PFP-16** (Figure 4F), we did not observe changes in PAX3::FOXO1 protein levels when the cells were treated with the PAX3::FOXO1 binder **PFB-a** (Figure S4A). This further supports the idea that the complete PROTAC molecule consisting of target binder, linker, and E3 ligase ligand is required for the compound’s function, and that **PFB-a** alone is not able to induce protein degradation. To further assess selectivity, we tested **PFP-16** in the Ewing sarcoma cell line TC71, which harbors the EWS::FLI1 fusion transcription factor rather than PAX3::FOXO1. Treatment with **PFP-16, PFP-4**, or **PFB-a** did not alter FOXO1 or EWS::FLI1 protein levels in TC71 cells (Figure S4B), indicating that **PFP-16** does not indiscriminately degrade transcription factors. Our data suggest that **PFP-16** does not degrade FOXO1 protein, which may indicate that **PFP-16** binds to the PAX3 side of the fusion protein. To address whether **PFP-16** degrades PAX3, we transiently overexpressed PAX3 in RH28 and TC71 cells and subsequently treated the cells with DMSO or **PFP-16** for 24 h. We used an overexpression system since the PAX3 antibodies that we have tested were unable to detect the low levels of endogenous PAX3 in our cell lines. We observed a robust decrease in the exogenous PAX3 protein levels following **PFP-16** treatment (Figure S4C), suggesting that **PFP-16** degrades PAX3, inferring that **PFP-16** binds PAX3::FOXO1 on the PAX3 side.

PAX3::FOXO1 expression changes between different phases of the cell cycle with an upregulation in G_2_ phase [67]. Therefore, it is possible to achieve indirect reduction in PAX3::FOXO1 protein levels by simply altering the cell cycle distribution of FP-RMS cells. To rule out this non-specific effect, we treated RH41 and RD cells with **PFP-16** or **PFP-4** for 24 h and performed cell cycle analysis via propidium iodide staining and flow cytometry. We did not observe any significant changes in cell cycle phases upon PFP treatment (Figure S4D). Taken together, these results further support that **PFP-16** is acting directly on the PAX3::FOXO1 protein.

### 3.3. PAX3::FOXO1-Targeting PROTAC Induces PAX3::FOXO1 Ubiquitination and Degradation via the Proteasome

**Figure 5:**
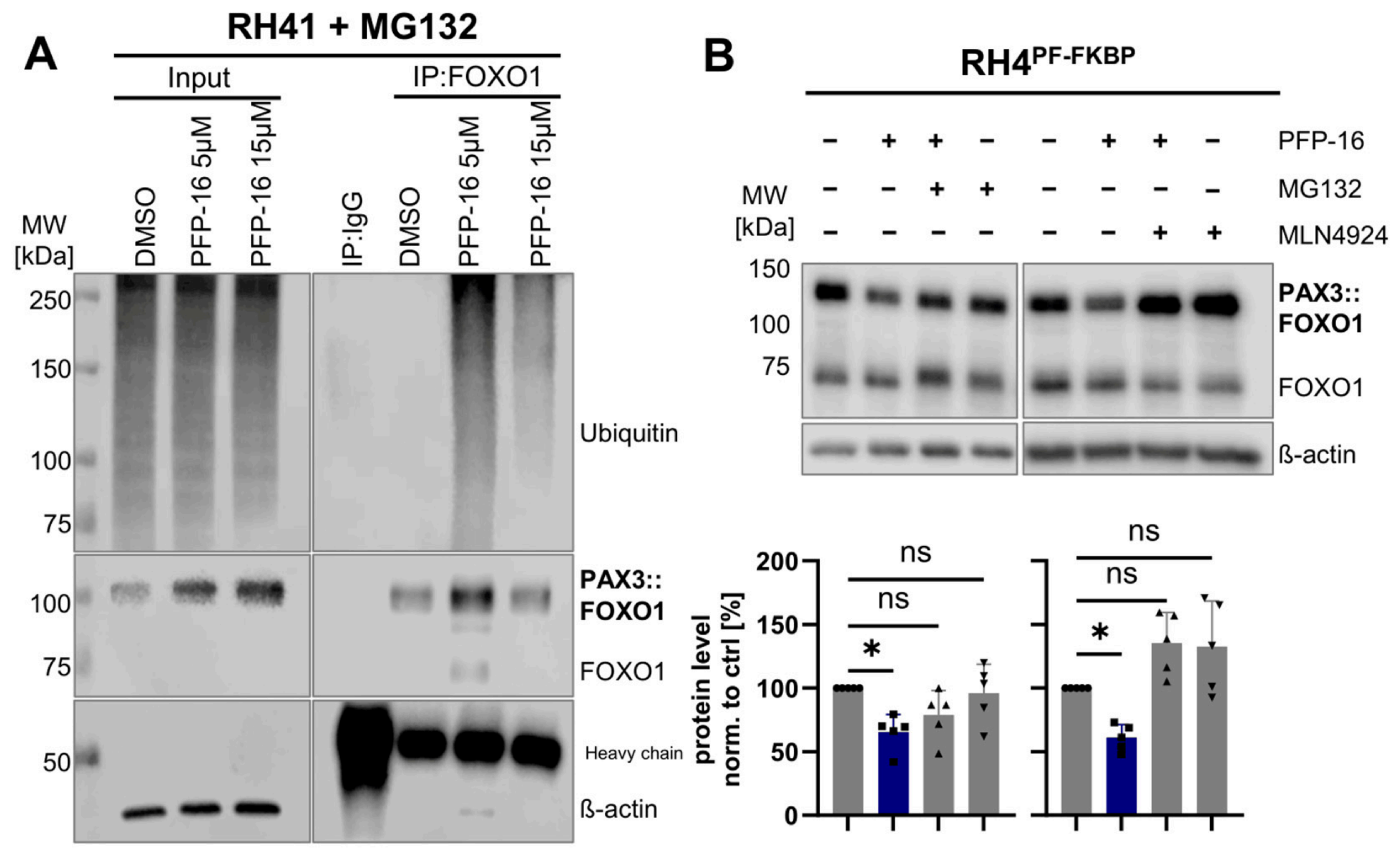
PAX3::FOXO1-targeting PROTAC induces PAX3::FOXO1 ubiquitination and degradation via the proteasome. (**A**) RH41 cells were pre-treated for 1 h with 300 nM proteasome inhibitor MG132 followed by **PFP-16** treatment or DMSO for 5 h. FOXO1 IP and western blot staining for ubiquitin, PAX3::FOXO1, and beta-actin loading control are shown. A representative image of three independently performed experiments is provided. (**B**) RH4^PF-FKBP^ cells pre-treated for 1 h with 1 µM proteasome inhibitor MG132 or 1 µM NEDD8-activating enzyme inhibitor MLN4924, followed by 24 h of 10 µM **PFP-16** treatment in the continued presence of MG132 or MLN4924. Western blot showing PAX3::FOXO1 and beta-actin as loading control with corresponding quantification (n = 5). Data presented as means +/− SD using one-Way ANOVA with Dunnett multiple testing correction, each condition compared to DMSO control. ns > 0.05, * *p* < 0.05.

To determine whether **PFP-16** degrades PAX3::FOXO1 protein on-mechanism, we tested whether **PFP-16** treatment results in the ubiquitination of PAX3::FOXO1 protein and subsequent proteasomal degradation. For this, we immunoprecipitated PAX3::FOXO1 protein following **PFP-16** treatment and immunoblotted for ubiquitin. We observed little to no ubiquitin signal in the DMSO-treated samples but a strong ubiquitin signal in the **PFP-16**-treated samples, indicating (poly-) ubiquitination events on PAX3::FOXO1 protein (Figure 5A). Moreover, using the proteasome inhibitor MG132 or NEDD8-activating enzyme inhibitor MLN4924, we were able to stabilize PAX3::FOXO1 protein and prevent its **PFP-16**-induced degradation (Figure 5B). Since Cullin-RING E3 ligase activity is activated via NEDD8 modification [68], inhibition of the enzyme that catalyzes the attachment of NEDD8 onto Cullin-RING E3 ligases such as SKP1 inactivates E3 ligase function and, with that, **PFP-16** activity. Thus, **PFP-16** requires an active Cullin-RING E3 ligase such as SKP1 to polyubiquitinate the PAX3::FOXO1 protein, which is then degraded via the proteasome.

### 3.4. PAX3::FOXO1-Targeting PROTAC Deregulates PAX3::FOXO1 Target Gene Expression and Induces Myogenic Differentiation in FP-RMS Cells

**Figure 6:**
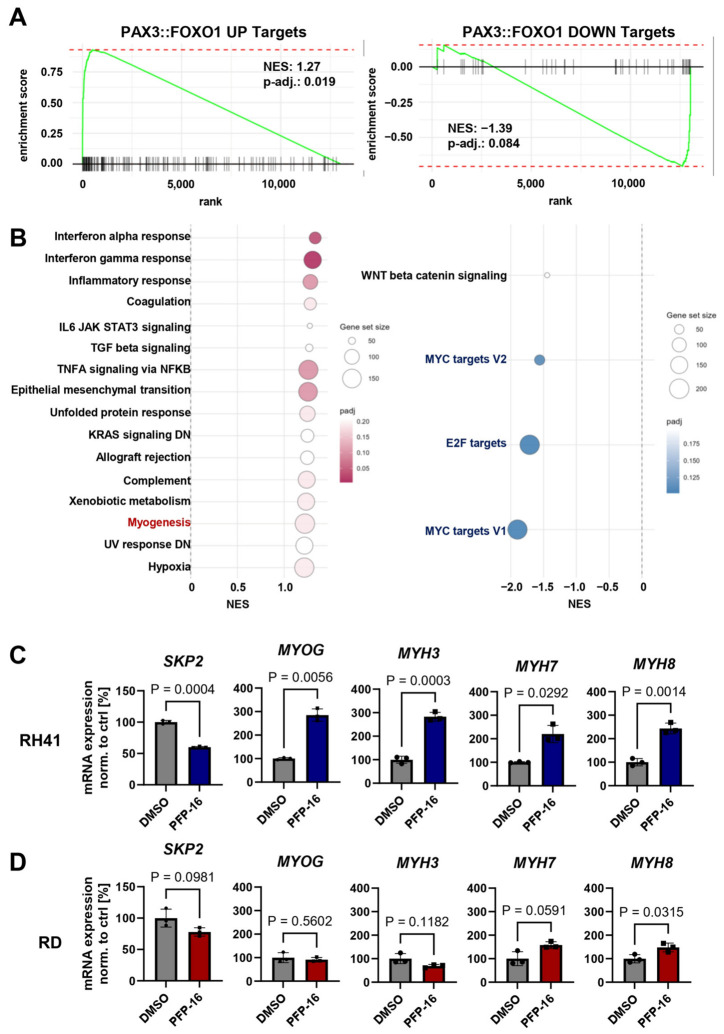
**PFP-16** impairs PAX3::FOXO1 target gene expression in FP-RMS cells. (**A**) Bulk RNA-sequencing analysis of RH41 cells treated with DMSO or 5 µM **PFP-16** for 7 days. GSEA for PAX3::FOXO1 target genes curated by Ebauer et al. [16] showing enrichment in **PFP-16**-treated cells. (**B**) GSEA using hallmark gene sets showing enrichment in **PFP-16**-treated cells with *p* adj. < 0.25. (**C**,**D**) qRT-PCR in RH41 (**C**) or RD (**D**) cells treated for 7 days with DMSO or 5 µM **PFP-16**. Gene expression normalized to *18S* gene and DMSO. Unpaired two-tailed *t*-test with Welch correction (n = 3).

Having established that **PFP-16** degrades PAX3::FOXO1 protein in a concentration-, time-, and proteasome-dependent manner, we next determined the biological significance of the PROTAC-mediated degradation of PAX3::FOXO1 in FP-RMS cells. As a fusion transcription factor, PAX3::FOXO1 is known to rewire the transcriptome with numerous target genes identified [14]. Therefore, we determined the changes in the transcriptome of RH41 cells treated with **PFP-16** by RNA-sequencing. GSEA with comparison to published PAX3::FOXO1 gene silencing studies revealed that **PFP-16** treatment deregulated PAX3::FOXO1 target gene expression signatures (Figure 6A) [16]. Moreover, analysis of hallmark gene sets revealed enrichment of myogenesis in PROTAC-treated cells as well as downregulation of PAX3::FOXO1-related pathways MYC and E2F targets (Figure 6B). Myogenic differentiation upon PAX3::FOXO1 loss has been described extensively in the literature, where inactivation of PAX3::FOXO1 led to induction of myogenic markers such as *myogenin (MYOG)* and *myosin heavy chain (MYH)* genes [39,64,69,70,71]. Seven-day **PFP-16** treatment significantly increased *MYOG*, *MYH3*, *MYH7*, and *MYH8* mRNA expression with only minor changes in *PAX3::FOXO1* or *SKP1* mRNA levels in RH41 cells (Figure 6C and Figure S5A). Moreover, *SKP2* mRNA levels were significantly decreased in **PFP-16**-treated cells (Figure 6C), consistent with the literature describing *SKP2* as a PAX3::FOXO1 target gene [72,73]. We did not observe pronounced changes in gene expression of these targets in FN-RMS RD cells (Figure 6D and Figure S5B).

**Figure 7:**
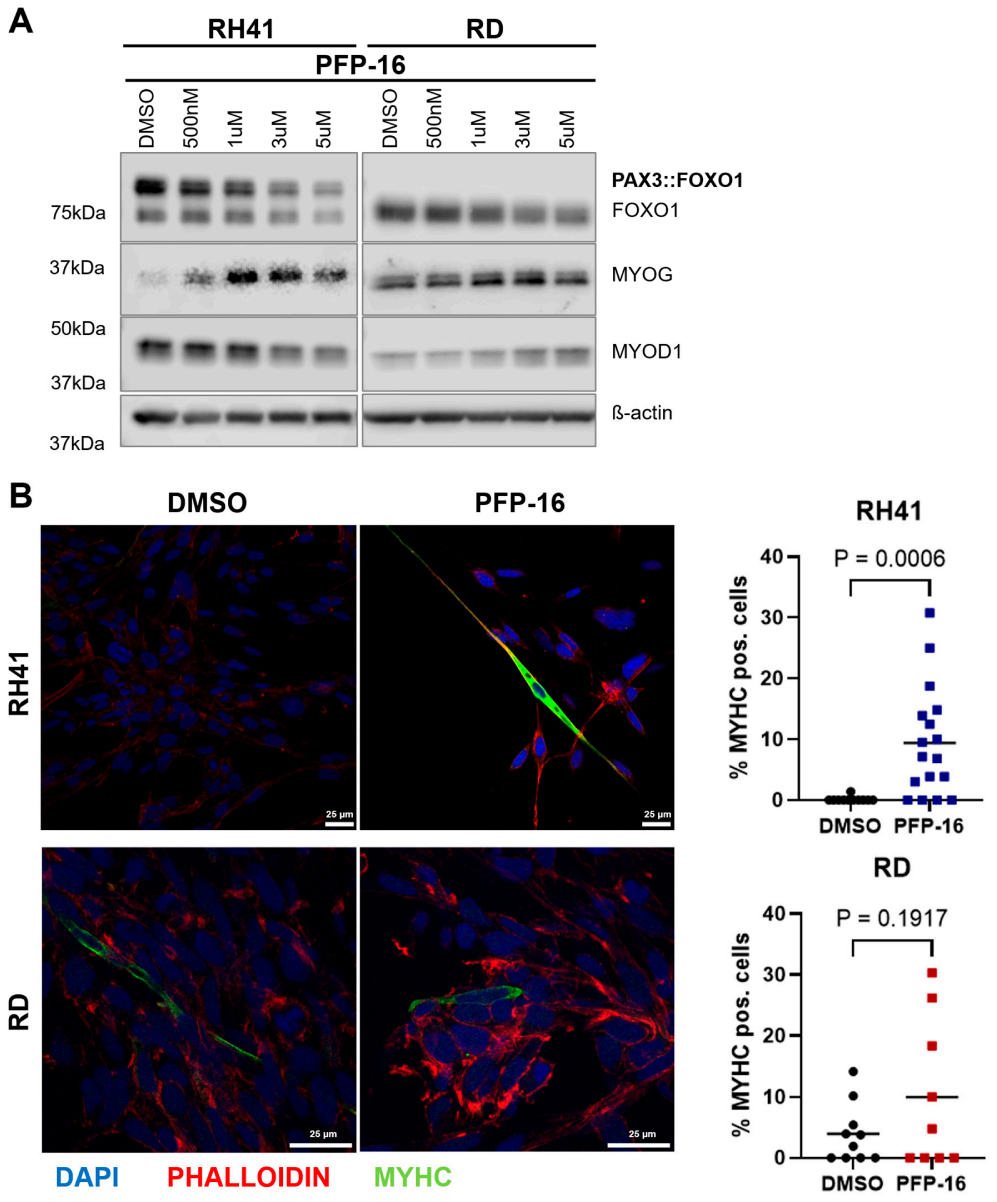
**PFP-16** induces myogenic differentiation in FP-RMS cells. (**A**) Western blot of myogenic marker MYOG, PAX3::FOXO1 target gene MYOD1, PAX3::FOXO1, and actin as a control in RH41 and RD cells. Cells were treated over 7 days with DMSO or **PFP-16** (RH41 n = 5, RD n = 3). (**B**) Immunofluorescence images acquired following the staining for myosin heavy chain, phalloidin, and DAPI in FP-RMS RH41 cells or FN-RMS RD cells following 7-day treatment with 5 µM **PFP-16**. Quantification of immunofluorescence images indicating % MYHC-positive cells within each acquired image shown as data points in the graph (RH41 DMSO n = 1286 cells; PFP-16 n = 435 cells and RD DMSO n = 550 cells; PFP-16 n = 308 cells). Unpaired two-tailed *t*-test with Welch correction.

To validate that PROTAC-treated FP-RMS cells undergo myogenic differentiation, we determined myogenic marker expression at the protein level as well. With decreasing PAX3::FOXO1 levels following **PFP-16** treatment, we observed an increase in MYOG levels and a decrease in PAX3::FOXO1 target MYOD1 levels in FP-RMS cells but not in FN-RMS cells (Figure 7A). Moreover, we used immunofluorescence staining for MYHC (myosin heavy chain) as a marker for terminal differentiation. FP-RMS RH41 cells showed a significant increase in MYHC levels following **PFP-16** treatment, which was not observed in FN-RMS RD cells (Figure 7B). Taken together, the degradation of PAX3::FOXO1 protein with **PFP-16** in FP-RMS cells deregulated PAX3::FOXO1 target gene expression and resulted in myogenic differentiation.

### 3.5. PAX3::FOXO1-Targeting PROTAC Synergizes with Vincristine Treatment and Impairs Anchorage-Independent Growth of FP-RMS Cells

**Figure 8:**
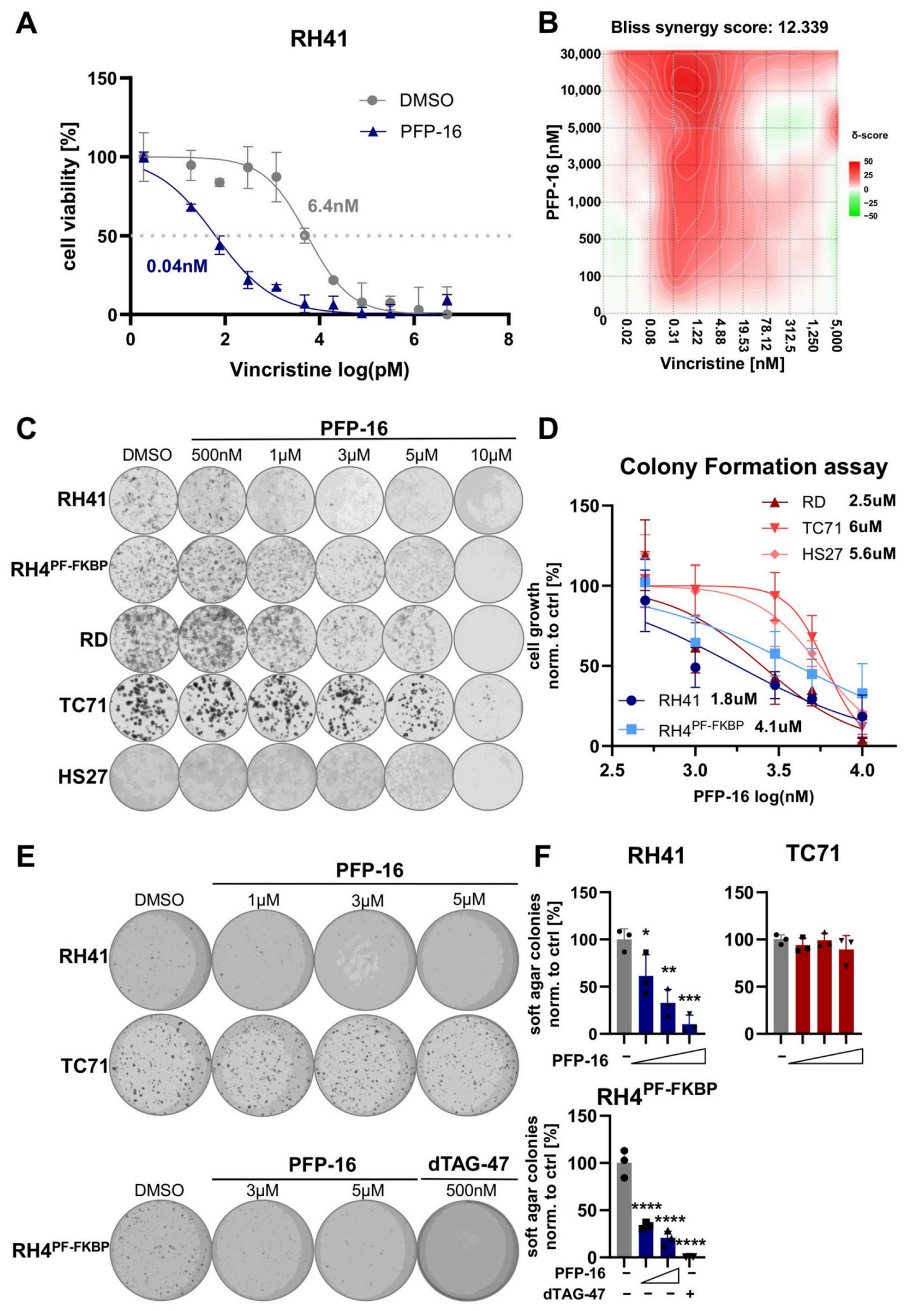
**PFP-16** synergizes with vincristine and impairs anchorage-independent growth in FP-RMS. (**A**) RH41 cells were treated with a concentration series of vincristine for 48 h in the presence or absence of 10 µM **PFP-16**, and subsequently cell viability was assessed using CellTiter-Blue reagent. Representative images of two independently performed experiments are shown with IC_50_ values displayed in the graph as the mean of the two experiments. (**B**) Drug synergy matrix obtained from combination treatments of **PFP-16** and vincristine for 48 h as in (**A**) using the Synergyfinder webtool (n = 1). (**C**) Colony formation assay and crystal violet staining of cells treated with increasing concentrations of **PFP-16** for 7d (TC71) or 10d (other cell lines) with corresponding quantification. (**D**) Representative image shown of four biological replicates. Drug response curve and IC_50_ values shown. (**E**) Anchorage-independent growth assessed using soft agar assay in TC71, RH41, and RH4^PF-FKBP^ cells. Cells were treated with increasing concentrations of **PFP-16** over 10d (TC71) or 21d (RH41, RH4^PF-FKBP^). (**F**) Quantification of soft agar images in E with data presented as means +/− SD and only significant changes displayed (n = 3). One-Way ANOVA with Dunnett multiple testing correction; only significant changes are displayed. * *p* < 0.05, ** *p* < 0.01, *** *p* < 0.001, **** *p* < 0.0001.

Previous studies have shown that KDM4 inhibition by entinostat, which decreases PAX3::FOXO1 expression, synergizes with vincristine treatment in FP-RMS cells [19,22]. Therefore, we hypothesized that **PFP-16** would also synergize with vincristine in FP-RMS cells. Indeed, the presence of **PFP-16** sensitized RH41 cells to vincristine from a half-maximal inhibitory concentration (IC_50_) of 6.4 +/− 0.9 nM in the DMSO control to 0.04 +/− 0.02 nM in the presence of 10 µM **PFP-16** (Figure 8A). Moreover, the combination treatment resulted in a synergistic score of 12.3 calculated via the Bliss model (scores > 10 are considered synergistic) using the webtool Synergyfinder (Figure 8B) [41]. Surprisingly, we also observed sensitization of **PFP-16** to vincristine in FN-RMS RD cells as well, with IC_50_ values of 3.51 +/− 2.69 nM in the DMSO control cells and 0.14 +/− 0.06 nM in the presence of **PFP-16** (Figure S6A). The Bliss score for this combination in RD cells was in the high additive range with a score of 8.2 (Figure S6B). However, the sensitization effect of **PFP-16** to vincristine treatment is more pronounced in FP-RMS RH41 cells compared to RD cells, with 160- and 25- fold changes in IC_50_ values, respectively.

In our experience, inhibition of PAX3::FOXO1 protein expression by siRNA or blocking its transcriptional activity using piperacetazine does not significantly reduce viability of FP-RMS cells in the short-term (24–72 h) 2D culture conditions [31]. In line with this, we see no changes in cell growth after a 2-day treatment of 10 µM **PFP-16** (Figure S6C), at which dose we see robust PAX3::FOXO1 protein degradation (Figure 4C–F). However, long-term treatment of **PFP-16** in a colony formation assay did reduce growth of FP-RMS cells in a concentration-dependent manner consistent with dTAG-47 treatment in RH4^PF-FKBP^ cells (Figure 8C,D and Figure S6D) [39]. Notably, TC71 Ewing sarcoma and HS27 fibroblast cells required much higher doses of **PFP-16** to observe any growth impairment (Figure 8C,D). Moreover, using a soft agar assay to measure anchorage-independent growth, **PFP-16** impaired FP-RMS growth significantly but not TC71 Ewing sarcoma cells (Figure 8E,F). In summary, **PFP-16** treatment synergized with vincristine treatment and impaired anchorage-independent growth in FP-RMS cells.

## 4. Discussion

In this study, we report the first-in-class PAX3::FOXO1-targeting PROTACs and characterize the functional consequences of their use in FP-RMS cells. We demonstrated that SPR can be effectively utilized for PROTAC design. We employed SPR previously to identify small-molecule inhibitors of difficult-to-target fusion transcription factors, resulting in the discovery of the EWS::FLI1-targeting drug YK-4-279 [74] as well as piperacetazine as a PAX3::FOXO1 inhibitor [31]. Consistent with piperacetazine-mediated inhibition of PAX3::FOXO1 function [31], **PFP-16**-mediated degradation of PAX3::FOXO1 was also able to impair PAX3::FOXO1 target gene expression (Figure 6) and anchorage-independent growth in FP-RMS cells (Figure 8). To our knowledge, we report the first PAX3::FOXO1-targeting PROTAC; however, our **PFP-16** lead compound still requires optimization in its (1) potency, (2) drug-like properties, and (3) selectivity.

TPD strategies are promising treatment avenues in pediatric cancers such as sarcomas, which are often aggressive diseases that do not respond well to conventional therapeutic interventions, including targeted therapy [75,76,77]. Several pediatric cancers harbor chromosomal translocations giving rise to fusion proteins [78,79] but are otherwise characterized by few co-occurring genetic alterations [80,81,82,83,84,85]. Thus, tumor-specific fusion proteins are considered the driving oncogenic event and represent attractive therapeutic targets. PROTACs have been successfully designed against fusion proteins harboring kinase domains such as BCR-ABL [86,87], ALK fusion proteins NPM-ALK and EML4-ALK [88,89], as well as the TPM3-TRKA fusion protein [90].

In contrast to fusion proteins consisting of proteins with enzymatic domains to which inhibitors have been extensively reported, many fusion proteins in pediatric cancers, including PAX3::FOXO1 in FP-RMS, are transcription factors [78] and, thus, inhibitors or small-molecule binders are not readily available to be incorporated into PROTAC design. The most prominent targets for small-molecule-based PROTACs targeting transcription factors are hormone receptors, androgen receptor (AR), and estrogen receptor (ER) [47,48,91]. Moreover, available small molecules against STAT3, STAT5, and SMAD3 were successfully used for PROTAC design [92,93,94]. For the vast majority of transcription factors to which no ligand or small molecule binder is available, several innovative approaches have been described demonstrating how PROTACs can be designed to target them [34]. Most of these rely on incorporating oligonucleotides as transcription factor-recruiting moieties, including TF-PROTACs [95], O’PROTACs [96], and (oligo-)TRAFTACs [97,98], and therefore have, to date, limited translational potential.

Our PROTAC design was achieved via screening small molecules for direct binding to the PAX3::FOXO1 protein via SPR [31], which we further used to determine binding affinities of our PROTACs to its target (Figure 2A, Figure 4B and Figure S1). Binding affinities ranged in the low µM range for PFPs, which will need to be optimized to improve the therapeutic potential of PAX3::FOXO1-targeting PROTACs. Although the binding affinity (K_D_) of **PFP-16** to PAX3::FOXO1 protein was 25.2 µM (Figure 4B), we achieved robust degradation of the PAX3::FOXO1 protein below this concentration with a DC_50_ of 7.7 µM following 24 h treatment in RH41 cells (Figure 4C,D), possibly enabled via positive cooperativity [49], which is the subject of future studies. Since the SKP1 recruiter that we incorporated into our PROTAC design only binds to SKP1 in complex with its SCF members at the interface of SKP1 and the CUL1 F-box substrate receptor but not to SKP1 monomers [63], determining binding affinities to ternary complex formation was further complicated and is under current investigation.

Our initial CRBN-based PROTAC design resulted in modest degradation of endogenous PAX3::FOXO1 protein in FP-RMS cells (Figure 2B,C), which we were able to improve by selecting SKP1 as another E3 ligase (Figure 3A–D). We chose SKP1 as an alternative E3 ligase for PFP design due to its relatively high expression in FP-RMS cell lines and patient tumor samples. More importantly, *SKP1* is an essential gene across cancer cell lines (depmap.org, Figure 3D), which may limit the development of acquired resistance to the PROTAC by cancer cells [33,62]. Resistance mechanisms towards CRBN- and VHL-based PROTACs emerged via mutations in the E3 ligase loci themselves or downregulation of the utilized UPS machinery members such as CUL2 or E2 enzyme UBE2G1 [61,99,100,101,102]. Thus, PROTACs that are recruiting an essential E3 ligase such as SKP1 may be less prone to acquired resistance mechanisms.

SKP1 also has a functional connection to RMS biology. SKP1’s F-box substrate receptor *SKP2* is overexpressed in RMS and directly targets p27^Kip1^ and p57^Kip2^ for proteasomal degradation, thereby driving cell cycle progression and preventing myogenic differentiation in FN-RMS [103]. Moreover, PAX3::FOXO1 has been shown to positively regulate *SKP2* expression in FP-RMS [72,73], where SKP2 was suggested to degrade p27^Kip1^ protein, resulting in cell cycle progression [72]. In line with this, we observed a decrease in *SKP2* mRNA levels following PFP-16 treatment (Figure 6C). Our data do not suggest inhibition of SKP2 function following **PFP-16** treatment, as we did not observe myogenic differentiation in RD cells (Figure 6D and Figure 7A,B), which was reported by Pomella et al. using SKP2 inhibition in FN-RMS cells [103]. However, it should be further explored if, and to what extent, **PFP-16** is inhibiting SKP1-SCF function, and which F-box proteins are affected by **PFP-16**, which may provide alternative molecular mechanisms of action in RMS. Moreover, a BioID study investigating PAX3::FOXO1 interactors identified SKP2 to be in close proximity to PAX3::FOXO1 [39], further supporting the idea that the SCF^SKP2^ complex plays important roles in FP-RMS and possibly positively affects **PFP-16**-mediated recruitment of SKP1 to PAX3::FOXO1 as they co-exist in the same subcellular locations.

Our early-stage compound still possesses some unfavorable physicochemical properties that may limit its use for in vivo testing. In addition to medicinal chemistry approaches, there have been several innovative strategies reported to overcome some of the poor characteristics of PROTACs, including large molecular weight, high hydrophobicity, and limited bioavailability [104,105,106,107,108,109]. For example, diverse pro-drug designs [110,111] can be applied to PAX3::FOXO1 targeting PROTACs. Over 30 PROTACs are currently pursued in clinical trials [34,112], with vepdegestrant being the first PROTAC recently gaining FDA approval, further highlighting their potential as an emerging therapeutic modality, especially in diseases with oncogenes that have been historically difficult to target, such as PAX3::FOXO1 in FP-RMS.

## 5. Conclusions

PAX3::FOXO1-targeting lead PROTAC **PFP-16** provides a starting point from which medicinal chemistry approaches can be initiated to potentially optimize its potency, selectivity, and physicochemical properties to develop a more clinical-grade compound for extensive pre-clinical evaluation to determine the therapeutic potential of PAX3::FOXO1-targeting PROTACs in FP-RMS patients. Our functional validation of **PFP-16**-mediated impairment of PAX3::FOXO1 target gene expression programs and induction of myogenic differentiation demonstrates that **PFP-16** effectively inhibits PAX3::FOXO1 function in FP-RMS cells. Moreover, conducted growth assays suggest that **PFP-16** can impair FP-RMS growth in long-term colony formation assays as well as 3D anchorage-independent growth. Together with the synergistic combination of **PFP-16** treatment with vincristine, our data support a favorable therapeutic potential. New and effective treatment options are an unmet clinical need for FP-RMS patients, and PAX3::FOXO1-targeting PROTACs may provide a new and, as demonstrated in this study, feasible opportunity for future clinical studies.

## Figures and Tables

**Table 1:** Primers used for qRT-PCR analysis.

Target Gene	Forward Primer 5′–3′	Reverse Primer 5′–3′
*18S*	GTAACCCGTTGAACCCCATT	CCATCCAATCGGTAGTAGCG
*MYOG*	CATGGAGCTGTATGAGACATCC	CGACTTCCTCTTACACACCTTAC
*SKP1*	AAC ACC GAA CAC CAT GCC TT	GGA ACT GGG TCA TCA TCT CCT
*SKP2*	GTG GTA TCG CCT AGC GTC TG	GGC AAT CAC CCC TTG AGA CA
*RBX1*	GGC AGC GAT GGA TGT GGA TA	AGG GCT ACT GCA TTC CAC TTT
*CUL1*	ACC ACA GAG ATG CGG GTT TG	TCA CAG CAG CTC AGA AAG GA
*CRBN*	CGA AGG AGA TCA GCA GGA CG	CTT CCT CAC TCT CTG CAG GC
*PAX3::FOXO1*	CTC ACC TCA GAA TTC AAT TCG TC	CCA CCA AGA ACT TTT TCC AG
*MYH3*	CTTGTGGGCGGAGGTCTG	AGCAGCTATGCCGAACACTT
*MYH7*	CTTGAGTAGCCCAGGCACAG	TGAGGTCAAAAGGCCTGGTC
*MYH8*	ACCAAGAACCCAGAGAGTGG	GAAGGTAGGGAGCAGCTTCG

## Data Availability

RNA-sequencing data generated in this study are publicly available on GEO with accession number GSE332748.

## Supplementary Materials

The following supporting information can be downloaded at: https://www.mdpi.com/article/10.3390/cancers18152375/s1, Figure S1. CRBN-based PROTACs directly bind to PAX3::FOXO1 protein; Figure S2. Effects of CRBN-based PFPs on cell viability and PAX3::FOXO1 mRNA; Figure S3. PFP-16 degrades PAX3::FOXO1 in a time-dependent manner; Figure S4. PFP-16 demonstrates on-target function; Figure S5. PFP-16 treatment impairs PAX3::FOXO1 function in FP-RMS cells; Figure S6. Effects of PFP-16 treatment on cell growth; Table S1. PAX3::FOXO1 binders incorporated in PROTAC design; Table S2. Chemical structures with corresponding molecular weights of PROTACs.
