# Supplementary material for: PAX3::FOXO1-Targeting PROTAC Induces Myogenic Differentiation of Fusion-Positive Rhabdomyosarcoma Cells
Source: Cancers (Basel). 2026 Jul 23;18(15):2375. doi: 10.3390/cancers18152375 (PMC13465285; doi:10.3390/cancers18152375)
Supplement: Supplementary file 1 [file cancers-18-02375-s001.zip › cancers-4425491-supplementary.pdf]

Table S1: PAX3::FOXO1 binders incorporated in PROTAC design.
*During PROTAC synthesis efforts it was observed that the PFB-a derivatives are unstable. To overcome this problem, the 9,10-dihydroacridine ring in PFB-a was replaced with a phenoxazine ring.

<table>
<tr><th>PAX3::FOXO1 binder (PFB)</th><th>PAX3::FOXO1 PROTAC (PFP)</th><th>comment</th></tr>
<tr><td>PFB-a*</td><td>PFP-1
PFP-2
PFP-3
PFP-4
PFP-5
PFP-16</td><td rowspan="6">Derivative of piperacetazine</td></tr>
<tr><td>PFB-b</td><td>PFP-6</td></tr>
<tr><td>PFB-c</td><td>PFP-7</td></tr>
<tr><td>PFB-d</td><td>PFP-8</td></tr>
<tr><td>PFB-e</td><td>PFP-9</td></tr>
<tr><td>PFB-f</td><td>PFP-10</td></tr>
<tr><td>PFB-g</td><td>PFP-11
PFP-12
PFP-13
PFP-14
PFP-15</td><td>PFB binder did not inhibit PAX3::FOXO1 activity</td></tr>
</table>

Table S2: Chemical structures with corresponding molecular weight of PROTACs.

PROTAC name	Structure	Molecular weight
**PFP-1**	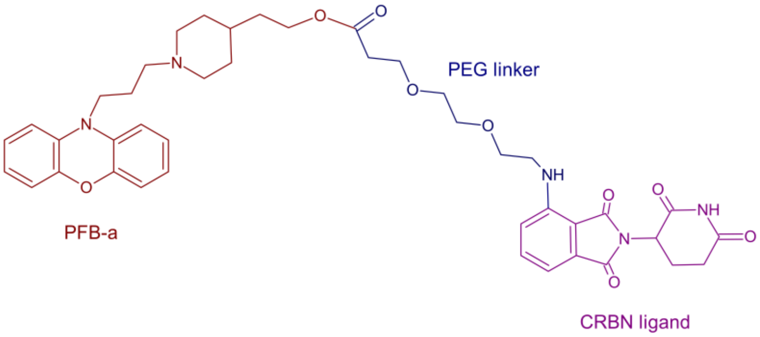	767.9 Da
**PFP-2**	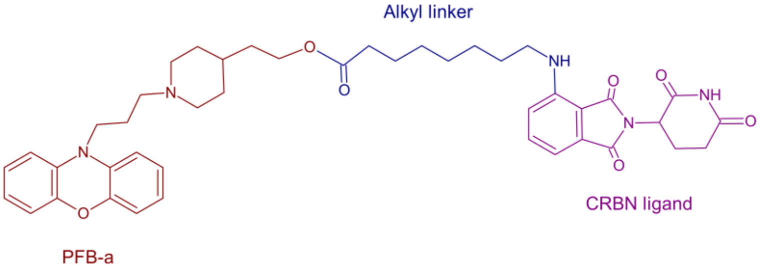	749.9 Da
**PFP-3**	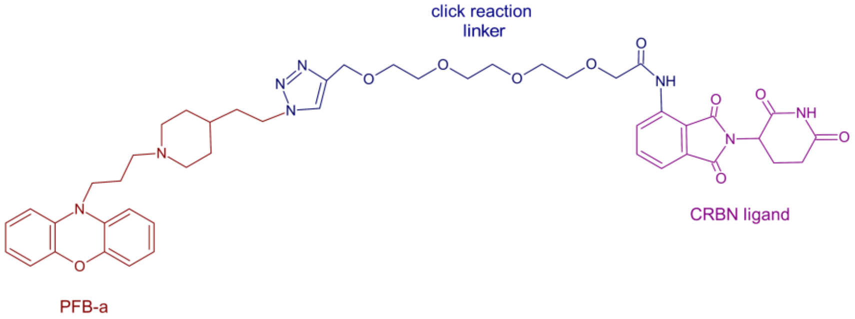	878.98 Da
**PFP-4**	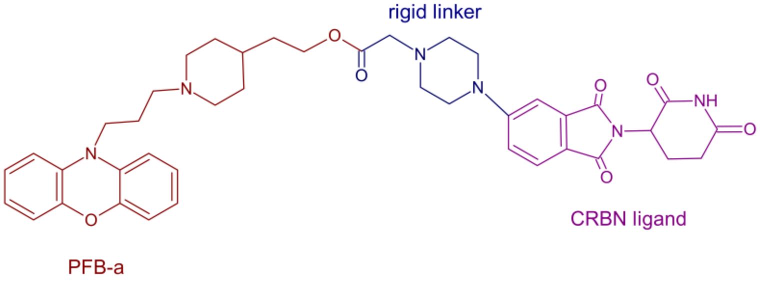	734.9 Da
**PFP-5**	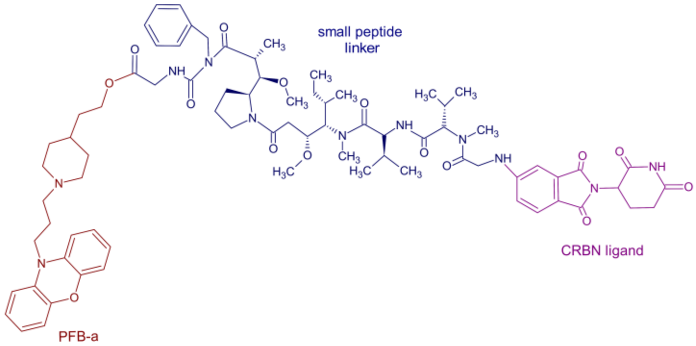	1436.76 Da

Table S2: Chemical structures with corresponding molecular weight of PROTACs.

PROTAC name	Structure	Molecular weight
**PFP-6**	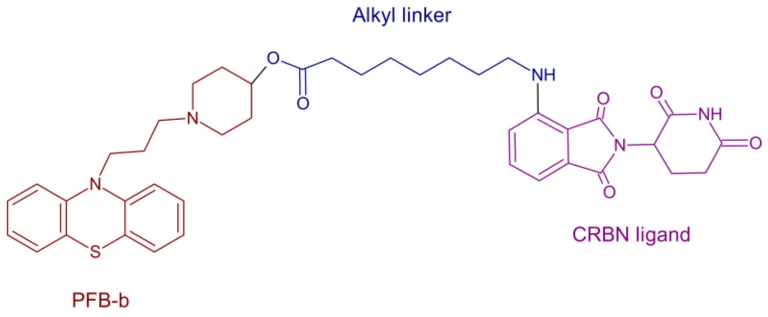	737.92 Da
**PFP-7**	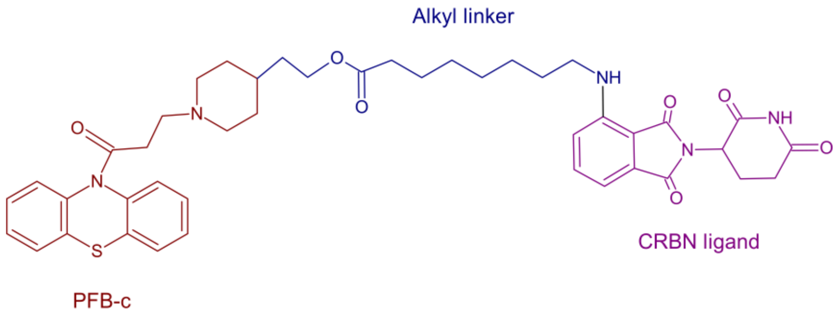	779.95 Da
**PFP-8**	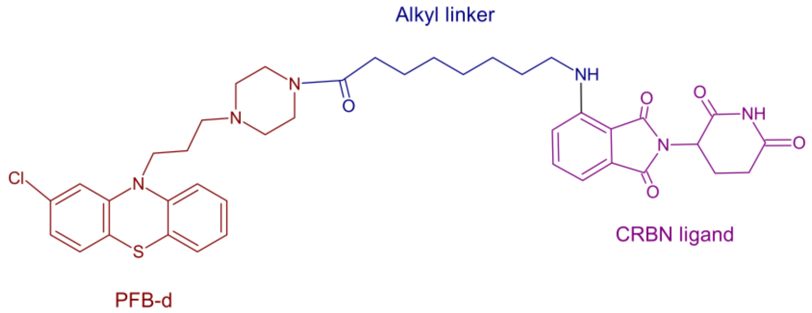	757.3 Da
**PFP-9**	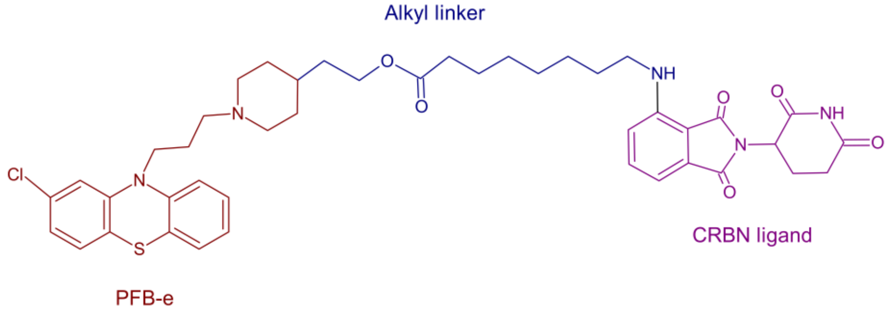	800.4 Da
**PFP-10**	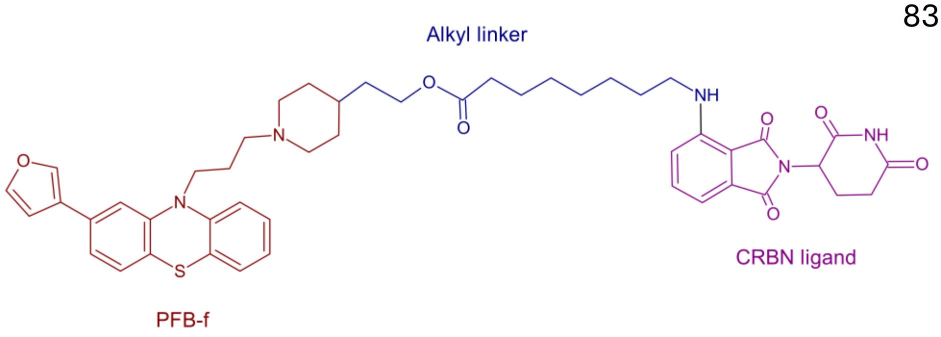	832.03 Da

Table S2: Chemical structures with corresponding molecular weight of PROTACs.

PROTAC name	Structure	Molecular weight
**PFP-11**	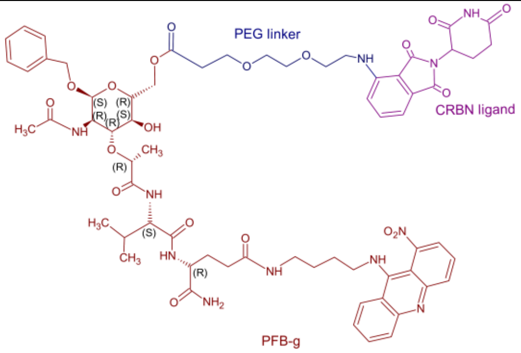	1318.4 Da
**PFP-12**	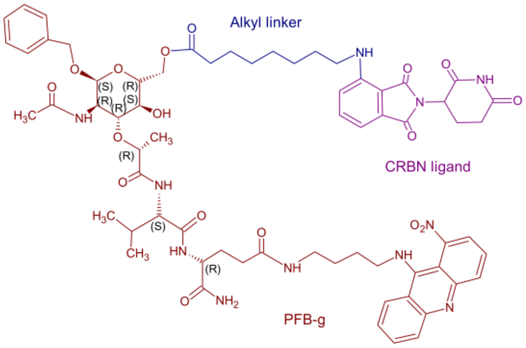	1300.4 Da
**PFP-13**	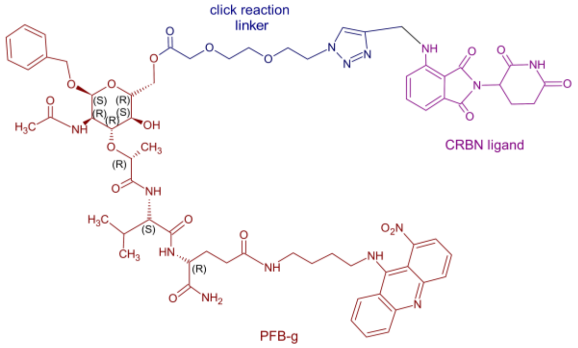	1385.4 Da
**PFP-14**	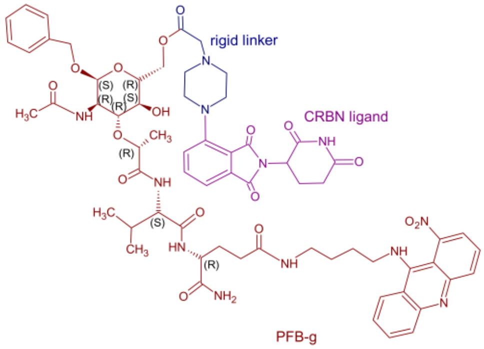	1285.4 Da
**PFP-15**	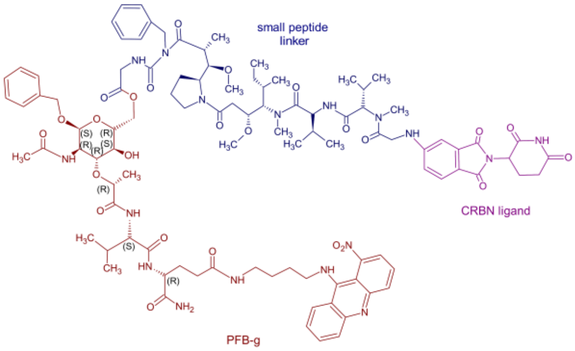	1987.3 Da

Figure S1: CRBN-based PROTACs directly bind to PAX3::FOXO1 protein.

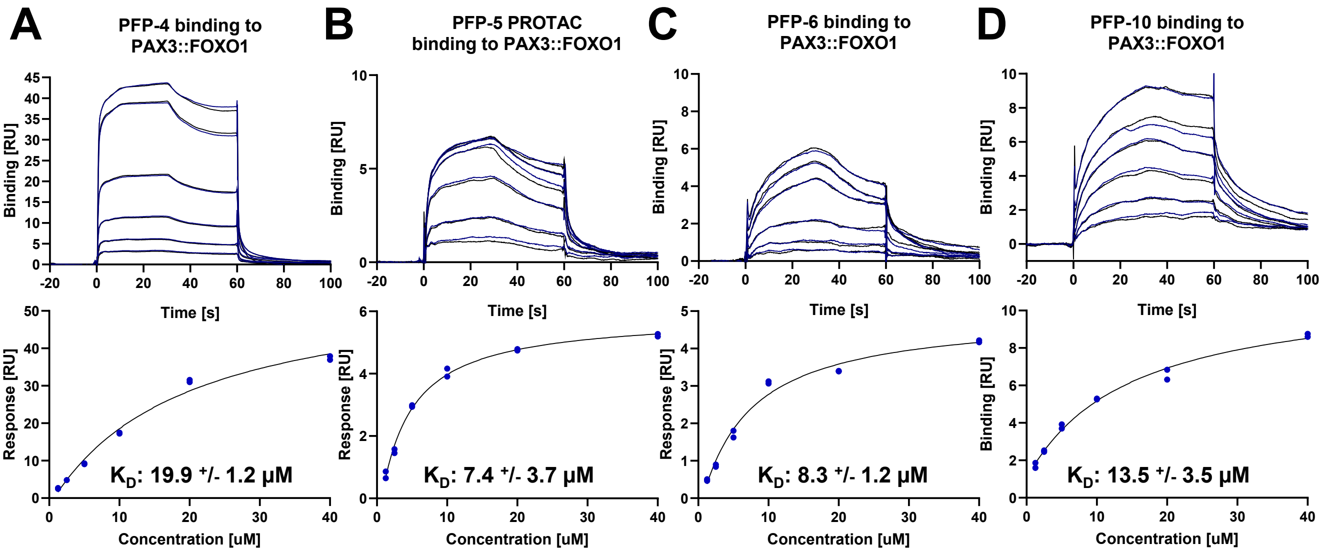

Legend: A-D) SPR sensorgrams for PROTACs PFP-4 (A), PFP-5 (B), PFP-6 (C), and PFP10 (D) demonstrate direct binding to PAX3::FOXO1 protein. PAX3::FOXO1 was immobilized on CM5 chip and PROTAC was used as the analyte at 6 different concentrations (40µM, 20µM, 10µM, 5µM, 2.5µM, and 1.25µM). KD values were determined via steady-state affinity fitting and are represented as mean and standard deviation (mean±s.d.) from three sets of SM-PAX3::FOXO1 bindings. Symbols correspond to response values towards the end of association signals (60s) and continuous lines represent the fit.

Figure S2: Effects of CRBN-based PFPs on cell viability and PAX3::FOXO1 mRNA.

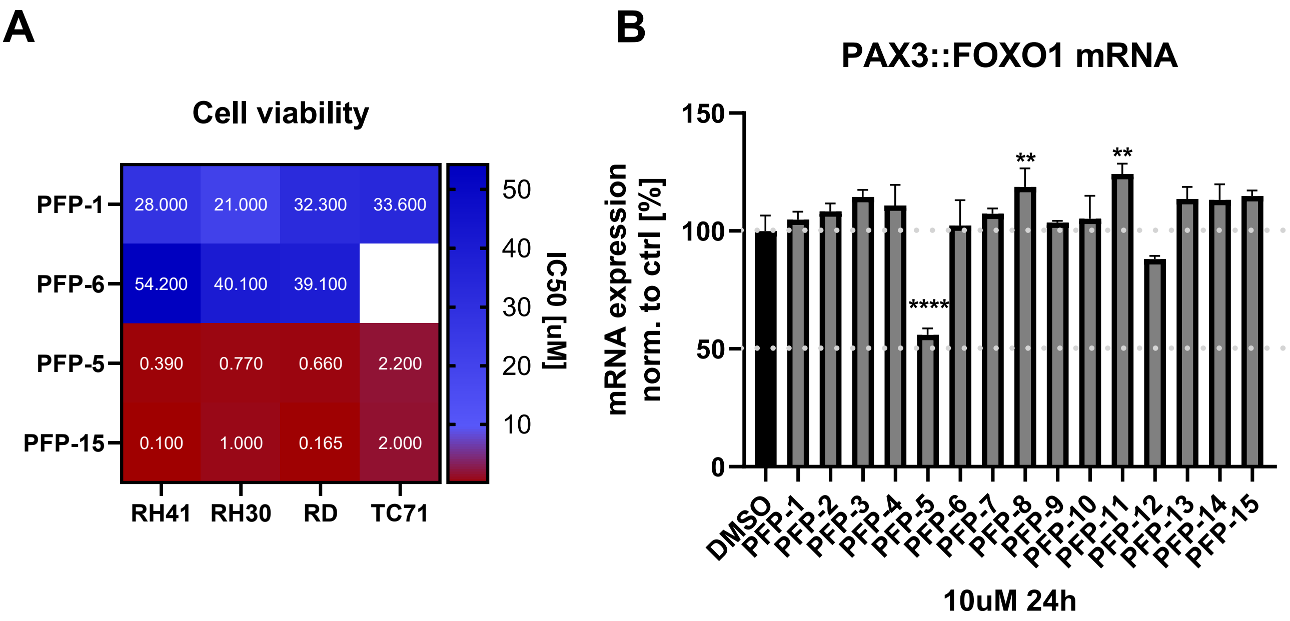

Legend: A) Cells were treated with a concentration series of PFPs for 48h and subsequently cell viability was assessed using CellTiter-Blue reagent. IC50 values displayed in heatmap. B) qRT-PCR of PAX3::FOXO1 mRNA in RH30 cells following 24h treatment of 10 µM PROTAC. PAX3::FOXO1 mRNA levels normalized to 18S and subsequently to DMSO. Data presented as means +/- SD and only significant changes displayed. One-Way ANOVA with Dunnett multiple testing correction each condition compared to DMSO treatment. *p < 0.05, **p < 0.01, ***p < 0.001, ****p < 0.0001.

Figure S3: PFP-16 degrades PAX3::FOXO1 in time-dependent manner.

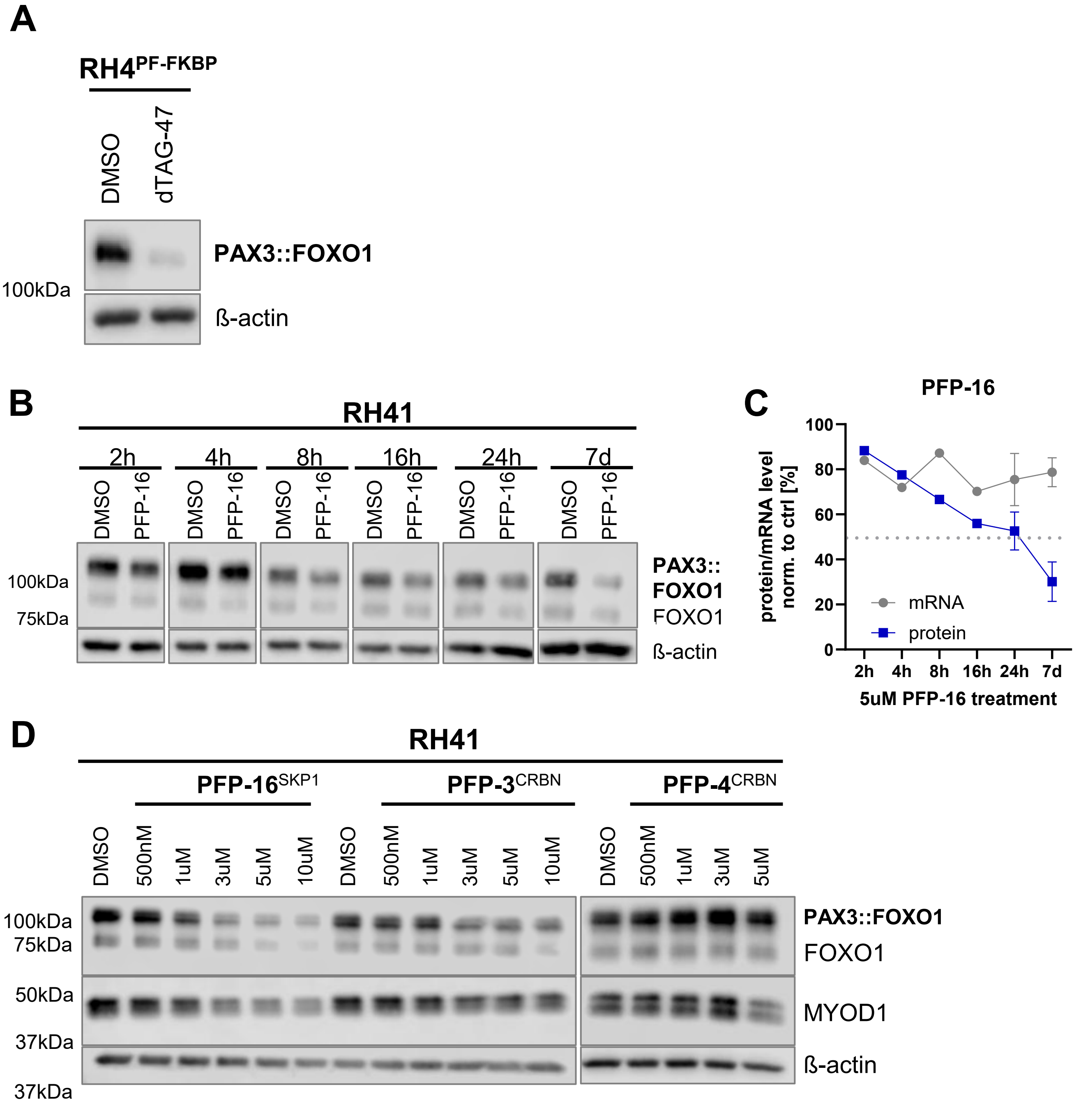

Legend: A) Western blot of RH4$^{PF\text{-}FKBP}$ cells treated for 24h with 500nM dTAG-47. B) Western blot of RH41 cells treated with 5 µM PFP-16 for indicated time course. C) Quantification of PAX3::FOXO1 protein level from B) and normalized to actin and subsequently corresponding DMSO control. At the same time points, qRT-PCR of PAX3::FOXO1 mRNA levels performed and normalized to 18S and subsequently DMSO control. D) Western blot of RH41 cells treated for 7 days with increasing concentrations of PFP-16, PFP-3, or PFP-4.

Figure S4: PFP-16 demonstrates on-target function.

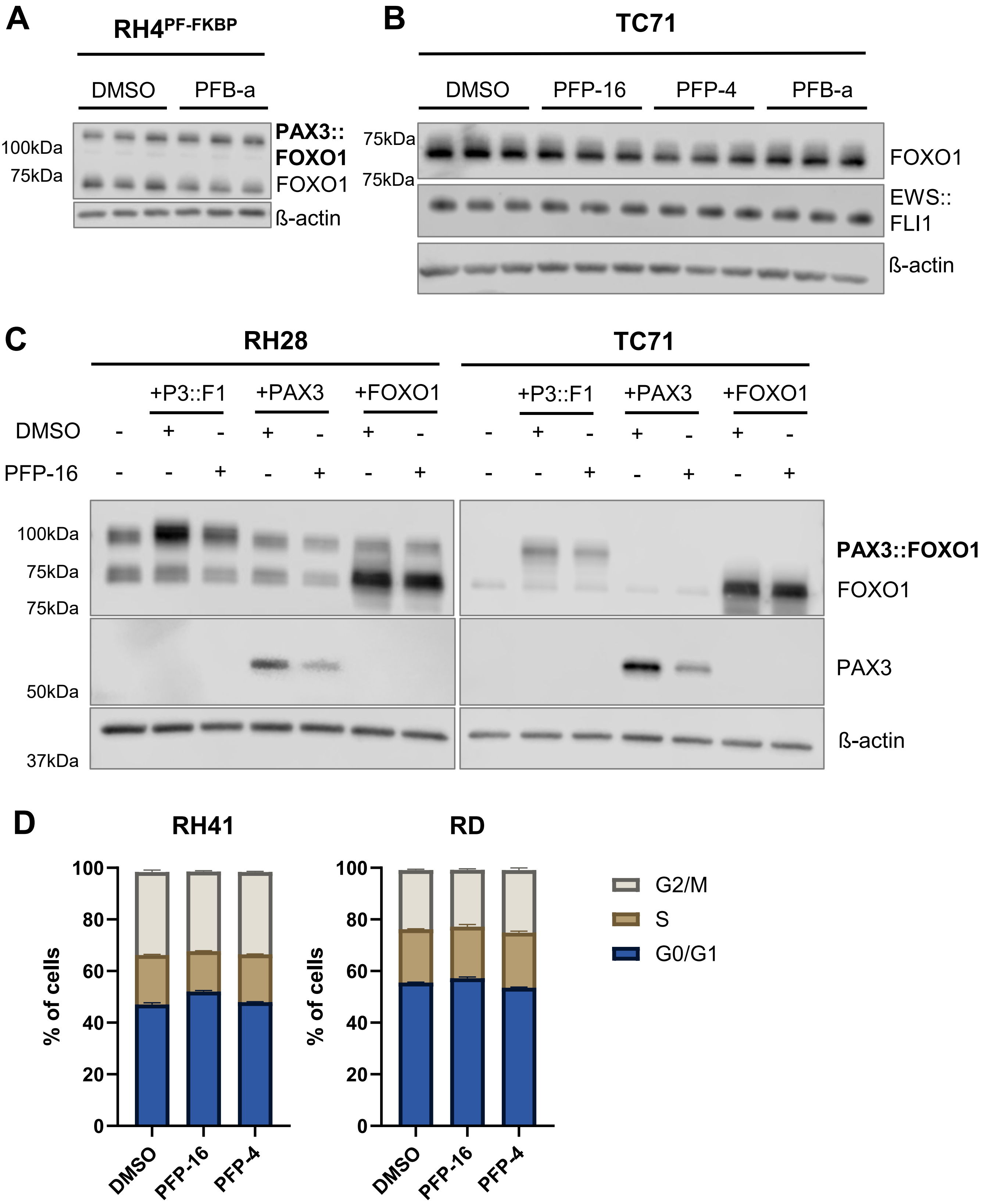

Legend: A) Western blot of RH4[PF-FKBP] cells treated for 24h with DMSO or 10 μM of PFB-a. B) Western blot of TC71 cells treated for 24h with 10 μM PFP-16, PFP-4, or PFB-a. C) Western blot of RH28 and TC71 cells transiently transfected with PAX3::FOXO1 (P3::F1), PAX3, or FOXO1 and subsequently treated for 24h with DMSO or 15 μM PFP-16 (n=3). D)Cell cycle analysis of RH41 and RD cells treated for 24h with 5 μM PFP-16 or PFP-4 (n=3).

Figure S5: PFP-16 treatment impairs PAX3::FOXO1 function in FP-RMS cells.

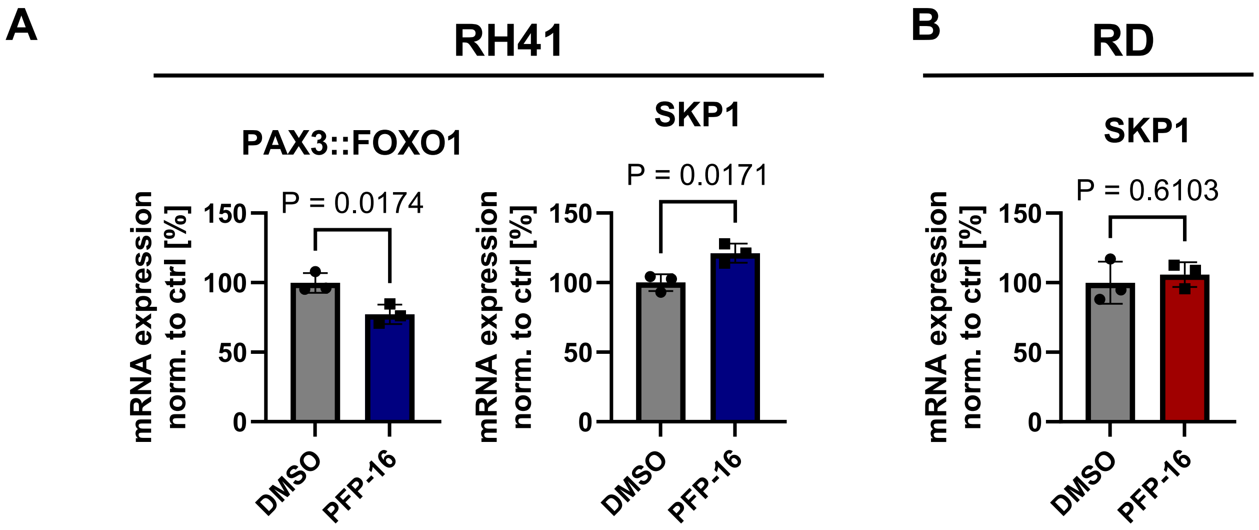

Legend: A, B) qRT-PCR of RH41 (A) or RD (B) cells treated for 7 days with DMSO or 5 µM PFP-16. mRNA expression normalized to 18S and subsequently to DMSO control. Unpaired two-tailed t-test with welch correction used (n=3).

Figure S6: Effects of PFP-16 treatment on cell growth.

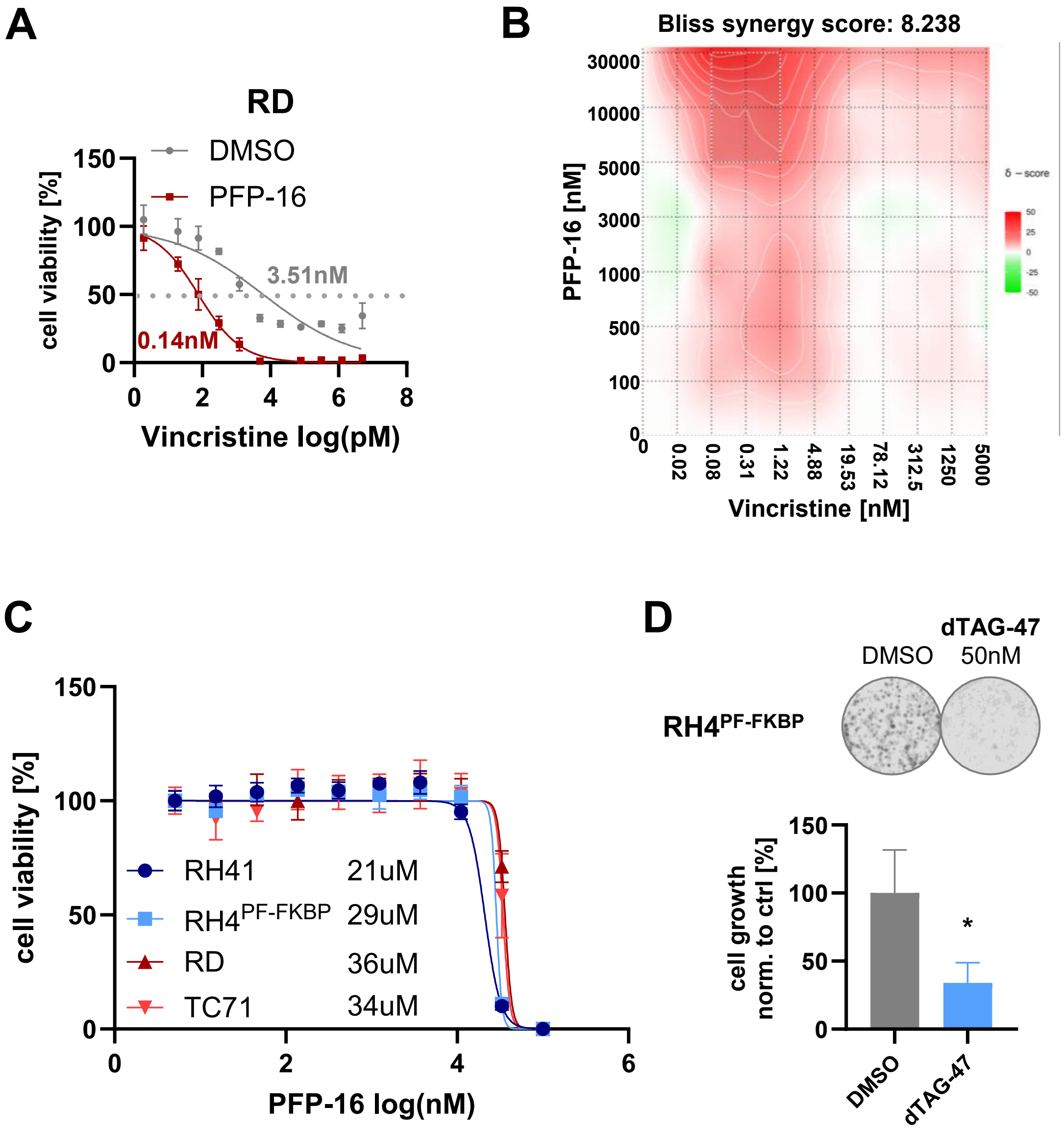

Legend: A) RD cells were treated with a concentration series of vincristine for 48h in the presence or absence of 10 µM PFP-16 and subsequently cell viability was assessed using CellTiter-Blue reagent. Representative images of 2 independently performed experiments shown (n=2) with IC50 values displayed in the graph as mean of the two experiments. B) Drug synergy matrix obtained from combination treatments of PFP-16 and vincristine for 48h as in A) (n=1). C) Cell viability of cell lines treated for 48h with concentration series of PFP-16 with IC50 values displayed in the graph. D) Colony formation assay and crystal violet staining of RH4$^{PF-FKBP}$ cells treated with 50nM of dTAG-47 for 10d with corresponding quantification. Representative image shown of 4 biological replicates. Unpaired two-tailed t-test with Welch correction. data presented as means +/- SD *$p < 0.05$.
